# Multi-omics analyses reveal ClpP activators disrupt essential mitochondrial pathways in triple-negative breast cancer

**DOI:** 10.3389/fphar.2023.1136317

**Published:** 2023-03-31

**Authors:** Emily M. J. Fennell, Lucas J. Aponte-Collazo, Wimal Pathmasiri, Blake R. Rushing, Natalie K. Barker, Megan C. Partridge, Yuan-Yuan Li, Cody A. White, Yoshimi E. Greer, Laura E. Herring, Stanley Lipkowitz, Susan C. J. Sumner, Edwin J. Iwanowicz, Lee M. Graves

**Affiliations:** ^1^ Department of Pharmacology and Lineberger Comprehensive Cancer Center, University of North Carolina at Chapel Hill, Chapel Hill, NC, United States; ^2^ Department of Nutrition, Nutrition Research Institute, University of North Carolina at Chapel Hill, Kannapolis, NC, United States; ^3^ Michael Hooker Proteomics Core Facility, University of North Carolina at Chapel Hill, Chapel Hill, NC, United States; ^4^ Women’s Malignancies Branch, National Cancer Institute, National Institutes of Health, Bethesda, MD, United States; ^5^ Madera Therapeutics, LLC, Cary, NC, United States

**Keywords:** ClpP activators, CLPP, mitochondria, multi-omics, breast cancer

## Abstract

ClpP activators ONC201 and related small molecules (TR compounds, Madera Therapeutics), have demonstrated significant anti-cancer potential in *vitro* and *in vivo* studies, including clinical trials for refractory solid tumors. Though progress has been made in identifying specific phenotypic outcomes following ClpP activation, the exact mechanism by which ClpP activation leads to broad anti-cancer activity has yet to be fully elucidated. In this study, we utilized a multi-omics approach to identify the ClpP-dependent proteomic, transcriptomic, and metabolomic changes resulting from ONC201 or the TR compound TR-57 in triple-negative breast cancer cells. Applying mass spectrometry-based methods of proteomics and metabolomics, we identified ∼8,000 proteins and 588 metabolites, respectively. From proteomics data, 113 (ONC201) and 191 (TR-57) proteins significantly increased and 572 (ONC201) and 686 (TR-57) proteins significantly decreased in this study. Gene ontological (GO) analysis revealed strong similarities between proteins up- or downregulated by ONC201 or TR-57 treatment. Notably, this included the downregulation of many mitochondrial processes and proteins, including mitochondrial translation and mitochondrial matrix proteins. We performed a large-scale transcriptomic analysis of WT SUM159 cells, identifying ∼7,700 transcripts (746 and 1,100 significantly increasing, 795 and 1,013 significantly decreasing in ONC201 and TR-57 treated cells, respectively). Less than 21% of these genes were affected by these compounds in ClpP null cells. GO analysis of these data demonstrated additional similarity of response to ONC201 and TR-57, including a decrease in transcripts related to the mitochondrial inner membrane and matrix, cell cycle, and nucleus, and increases in other nuclear transcripts and transcripts related to metal-ion binding. Comparison of response between both compounds demonstrated a highly similar response in all -omics datasets. Analysis of metabolites also revealed significant similarities between ONC201 and TR-57 with increases in α-ketoglutarate and 2-hydroxyglutaric acid and decreased ureidosuccinic acid, L-ascorbic acid, L-serine, and cytidine observed following ClpP activation in TNBC cells. Further analysis identified multiple pathways that were specifically impacted by ClpP activation, including ATF4 activation, heme biosynthesis, and the citrulline/urea cycle. In summary the results of our studies demonstrate that ONC201 and TR-57 induce highly similar and broad effects against multiple mitochondrial processes required for cell proliferation.

## Introduction

Metabolic reprogramming was first characterized in cancer cells in the 1920s following the identification of increased glycolytic dependence under aerobic conditions (Warburg et al., 1927; Warburg, 1956; Lunt and Vander Heiden, 2011). Known as the Warburg effect, aerobic glycolysis has been confirmed in many rapidly proliferating cell types, but is widely debated as cancer cells often retain functional mitochondria and exhibit sensitivity to mitochondria-targeting compounds (Darzynkiewicz et al., 1981; Zu and Guppy, 2004; Fogal et al., 2010; Lunt and Vander Heiden, 2011). It has been proposed that the observed upregulation in glycolysis serves to increase adenosine triphosphate (ATP) as well as production of glycolytic intermediates required for anabolic processes, including the tricarboxylic acid (TCA) cycle, amino acid, nucleotide, and lipid biosynthesis (Lunt and Vander Heiden, 2011; Deberardinis and Chandel, 2016; Vander Heiden and Deberardinis, 2017). Cancer dependencies on other metabolic processes have been identified, including glutaminolysis, serine biosynthesis, and nicotinamide adenosine dinucleotide (NAD^+^), and this hypothesis continues to be investigated (Vander Heiden and Deberardinis, 2017). The identification of metabolic pathways as vulnerabilities in cancer cells has led to the development of numerous metabolism-specific anticancer approaches (approved or in trials) which target mitochondrial metabolic pathways, including oxidative phosphorylation (OXPHOS) (e.g., metformin), TCA cycle (e.g., CPI-613), and glutaminolysis (e.g., CB-839) (Luengo et al., 2017; Vander Heiden and Deberardinis, 2017).

ONC201 was initially discovered in a TRAIL induction screen (Allen et al., 2015) and while showing anticancer efficacy in multiple cancer models, its mechanism of action was largely undefined when it entered into Phase I clinical trials (Allen et al., 2016; Ralff et al., 2017; Stein et al., 2017). Greer et al. were the first to demonstrate specific effects of ONC201 on mitochondrial function (Greer et al., 2018). Recently, ONC201 and its structural analogs (the TR compounds) were identified as highly specific activators of the mitochondrial protease ClpP (Graves et al., 2019; Ishizawa et al., 2019; Fennell et al., 2022). ClpP forms a complex with ClpX, an ATP-dependent unfoldase that facilitates proteins insertion into the tetradecameric core of ClpP where they are subsequently degraded (Bhandari et al., 2018; Wong et al., 2018; Mabanglo and Houry, 2022). The proteolytic activity of ClpP not only serves to maintain protein homeostasis within the mitochondria, but is known to induce the mitochondrial unfolded protein response (UPR^mt^) and integrated stress response (ISR), leading to further transcriptional regulation of metabolic and mitochondrial quality control-related genes (Haynes et al., 2007; Qureshi et al., 2017; Melber and Haynes, 2018; Anderson and Haynes, 2020; Kumar et al., 2022)*.* In mammalian cells, activating transcription factor 4 (ATF4) has been identified as the key regulator of ISR (Quirós et al., 2017), and we and others have shown ClpP-dependent induction of ATF4 in TNBC cells following ClpP activation (Fennell et al., 2022).

Activation of ClpP in multiple cancer cell models leads to non-specific degradation of mitochondrial proteins, including OXPHOS subunits (Ishizawa et al., 2019; Nguyen et al., 2022; Wang et al., 2022). We have shown the more potent activators of ClpP (TR compounds) result in depletion of mtDNA *in vivo*, as well as a ClpP-dependent loss of oxygen consumption rate (OCR) and protein-level expression of many mitochondrial metabolic, transcriptional, and homeostatic proteins in triple-negative breast cancer (TNBC) cells (Fennell et al., 2022). Further investigation into the mechanism of the TR compounds revealed a ClpP-dependent dysregulation of metabolic pathways including one-carbon metabolism, proline biosynthesis, and the mevalonate pathways in TNBC cells, leading to inhibition of breast cancer stem cell function (Greer et al., 2022).

The paradigm of the UPR^mt^ involves increased mito-nuclear signaling, protein-level changes, and metabolic remodeling (Haynes et al., 2007; Qureshi et al., 2017; Melber and Haynes, 2018; Anderson and Haynes, 2020). Consistent with this, multiple studies have reported protein- and transcript-level perturbations following ONC201 or TR compound treatment in multiple cancer models (Ishizawa et al., 2016; Kline et al., 2016; Greer et al., 2018; Graves et al., 2019; Ishizawa et al., 2019; Fennell et al., 2022; Greer et al., 2022; Nguyen et al., 2022). The dysregulation of mitochondrial metabolic activity, protein level, and transcriptomic profiles in a wide variety of cancer cell models indicates the need to investigate the full scope of perturbations occurring in cancer cells following ClpP activation. Multi-omics data analysis approaches are a valuable tool for interrogating pathways altered by drug exposures, genetic variation, and disease states (Ebrahim et al., 2016; Hasin et al., 2017). Utilization of a multi-omics approach to analyze the proteomic, transcriptomic, and metabolomic landscapes of TNBC cells treated with ONC201 or TR-57 provides the opportunity to achieve a broad understanding of cellular processes altered by ClpP activation.

The objective of this study was to complete a multi-omics profile of TNBC cells following pharmacological ClpP activation. We performed individual analyses of proteomic, transcriptomic, and metabolomic data, as well as multi-omics data analysis using MetaCore pathway analysis software. The data presented herein demonstrates a comprehensive analysis of proteomic, transcriptomic, and metabolomic perturbations in wildtype (WT) and ClpP knockout (ClpP-KO) TNBC cells. Our multi-omics analysis identified significant alterations in mitochondrial metabolic processes, including TCA cycle (S)-citrulline metabolism, amino acid metabolism, ATF4 induction, and heme biosynthesis.

## Results

### Multi-omics analyses reveals loss of mitochondria-specific functions following ClpP activation in TNBC cells

**FIGURE 1:**
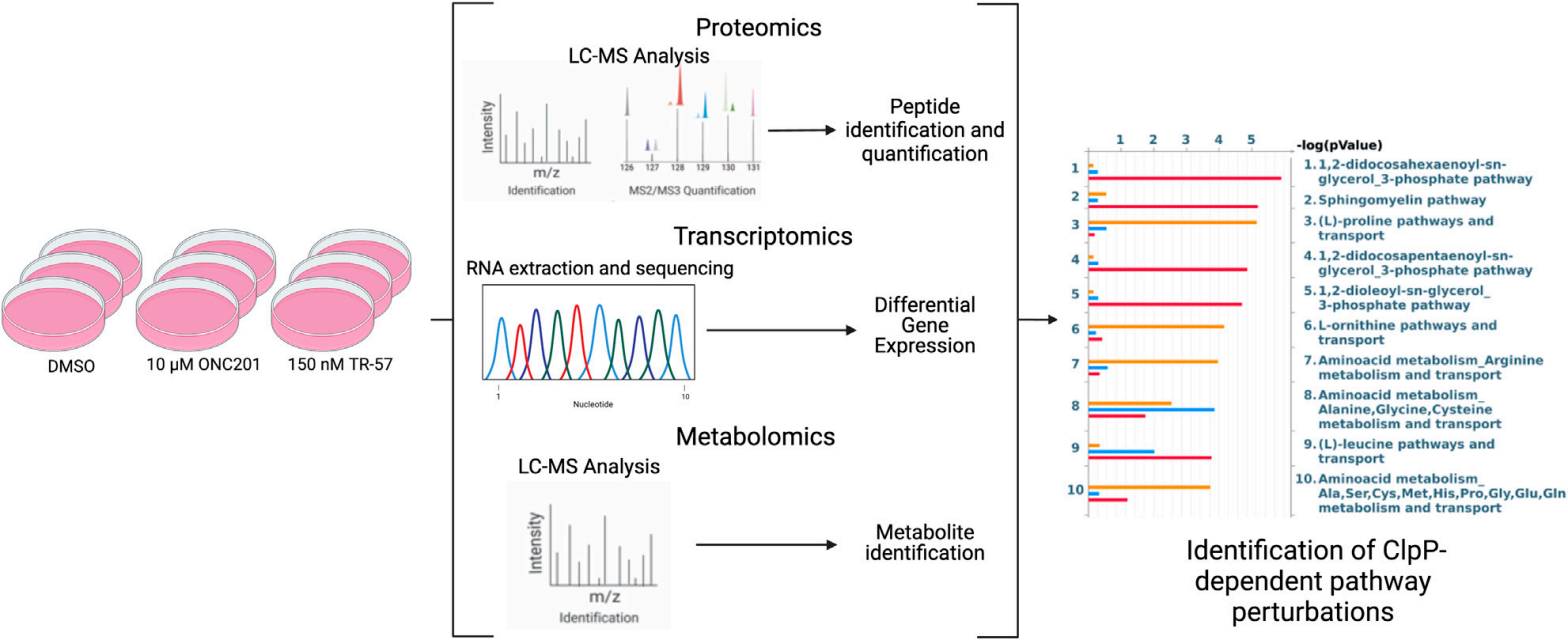
Schematic of multi-omics data collection and analysis of triple-negative breast cancer cells. SUM159 cells were treated with 0.1% DMSO, 10 µM ONC201, or 150 nM TR-57 and samples were collected and prepared for transcriptomics, metabolomics, and proteomics analyses using appropriate methods. Datasets were then analyzed individually using standard methods and as a combined multi-omics dataset using MetaCore. N = 3.

To gain a better understanding of the biological consequences of ClpP activation in cancer cells, we compared the effects of 2 well characterized ClpP activators in a model of TNBC. Wildtype SUM159 (WT) and SUM159 ClpP-knockout (ClpP-KO) cells were treated with 10 μM ONC201 or 150 nM TR-57 (∼10 fold higher than the respective IC_50_ value previously determined for each compound (Graves et al., 2019)) for 24 h. Following extraction, LC-MS analysis and protein/metabolite identification were performed to obtain the proteomics and metabolomics datasets as described in Methods. In parallel, RNAseq was performed on mRNA isolated from treated cells to obtain the transcriptomics data (Figure 1). From these studies, approximately 8,000 proteins and 7,700 transcripts were quantified in each condition (Supplementary Data S1,S4, respectively). Approximately 4,100 metabolites were measured, 3,218 which were matched to specific metabolites using an in-house library or public databases, and 588 were utilized for metabolomics analysis (ontology levels OL1, OL2a/2b, and PDa; see Materials and Methods and Supplementary Data S6). Additionally, phosphoproteomics analysis was performed on these samples, which yielded ∼17,000 phosphopeptides (Supplementary Figure S1, Supplementary Data S2).

#### Proteomics

**FIGURE 2:**
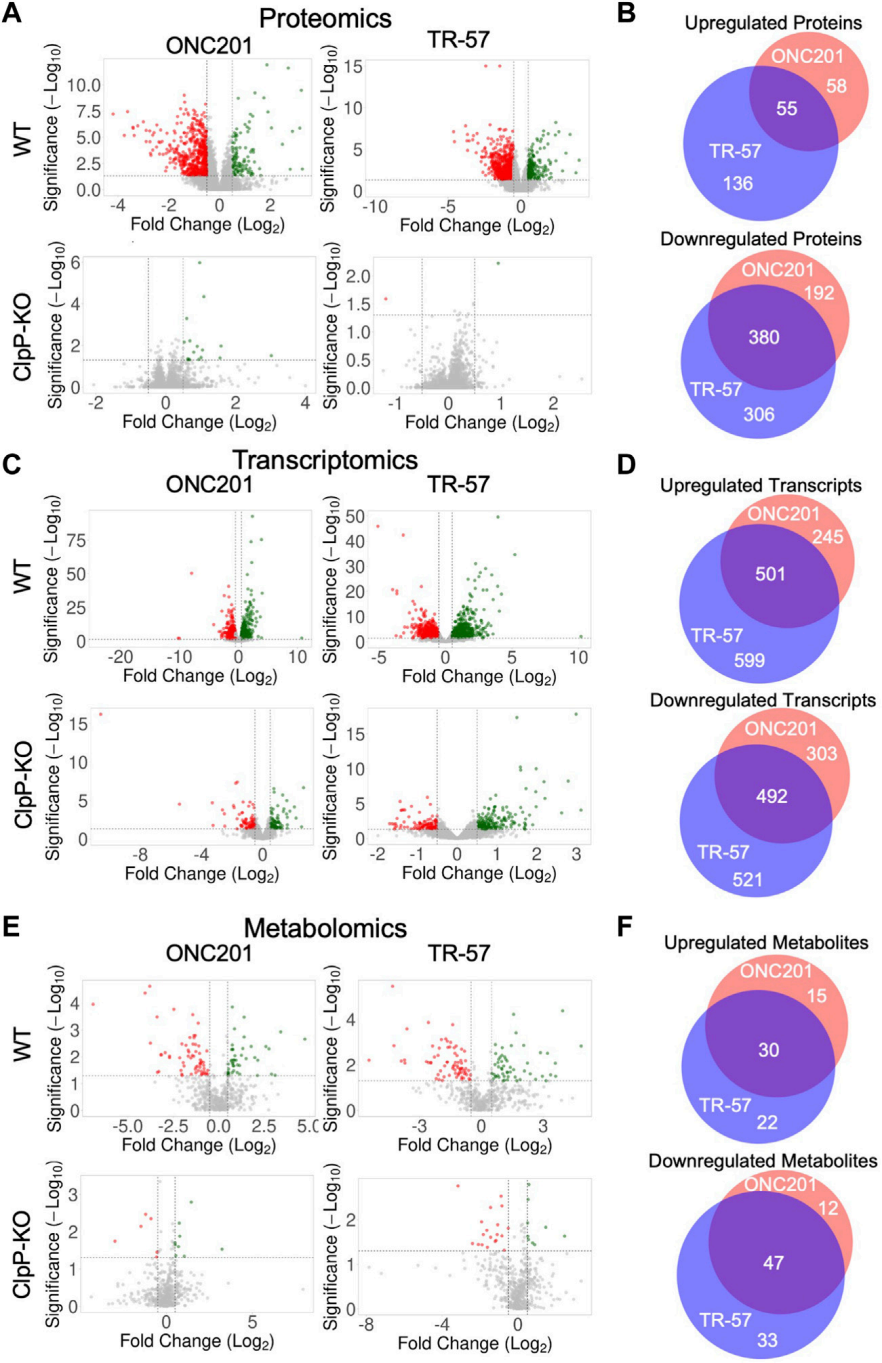
Analysis of omics data reveals significant and similar ClpP-dependent effects of ONC201 and TR-57 on triple-negative breast cancer cells. Wildtype (WT) and ClpP knockout (ClpP-KO) SUM159 cells were treated with 10 μM ONC201 or 150 nM TR-57 for 24 h and analyzed for proteomic **(A, B)**, transcriptomic **(C, D)**, and metabolomic **(E, F)** perturbations. Volcano plots of each dataset are shown in **(A, C)**, and **(E)**. Axes are Log_2_(Fold Change) and -Log_10_ (unadjusted *p*-value). Vertical dashed lines indicate fold change threshold (Log_2_(FC) >|0.5|) and horizontal dashed lines indicate significance threshold (-Log_10_ (*p*-value) >1.3), N = 3. Venn diagrams of significantly perturbed proteins **(B)**, transcripts **(D)**, and metabolites **(F)** were generated from small molecules meeting the fold change and significance thresholds previously mentioned. Red: ONC201; blue: TR-57; purple: Both drug treatments.

Analysis of the total proteomics revealed significant differences between WT and ClpP-KO SUM159 cells treated with either ONC201 or TR-57 (Figure 2A). Of the ∼8,000 proteins quantified, many proteins were significantly downregulated (Log_2_ (fold change) ≤ −0.5 and *p*-value ≤0.05) in ONC201 and TR-57 treated cells compared to DMSO control (572 and 686 proteins for ONC201 and TR-57 treated cells, respectively). In comparison, only 113 and 191 (ONC201 and TR-57, respectively) proteins were found to be significantly upregulated (Log_2_ (fold change) ≥0.5 and *p*-value ≤0.05). Overall ClpP activation with ONC201 or TR-57 influenced protein changes in a highly similar manner, with 55 upregulated and 380 downregulated proteins in common (Figure 2B). By contrast, very few proteins were significantly affected by either ONC201 or TR-57 treatment in ClpP-KO cells, suggesting the proteome changes observed in WT cells upon treatment are a direct result of ClpP activation. ONC201 treated ClpP-KO cells showed only 19 significantly upregulated and 0 significantly downregulated proteins, whereas TR-57 treated ClpP-KO cells showed only 1 significantly perturbed protein in either direction (Figure 2A).

Gene ontological (GO) analysis of this data indicated a significant downregulation in mitochondrial proteins as expected for activation of a mitochondrial matrix protease (Supplementary Figure S2A). In addition to proteins involved in mitochondrial translation, GO analysis of significantly downregulated proteins revealed enrichment in the oxidative phosphorylation pathway, consistent with previous data that ONC201 and TR compounds reduce OXPHOS in TNBC and other cells (Greer et al., 2018; Ishizawa et al., 2019; Fennell et al., 2022; Greer et al., 2022). Proteins significantly downregulated by ONC201 and TR-57 treatment included ATPase Inhibitor (ATPIF1), mitochondrial 39S ribosomal protein L12 (MRPL12), and mitochondrial elongation factor Tu (TUFM) (Supplementary Figure S2B). GO analysis of the upregulated proteins demonstrated increases in mainly nuclear, cytosolic, and membrane proteins following ClpP activation, including the proteins Niban 1 (NIBAN1), peroxisome proliferator-activated receptor delta (PPAR-δ), growth/differentiation factor 15 (GDF15) and others Supplementary Figures S2A,B).

Analysis of the phosphoproteomics dataset identified changes in protein phosphorylation and kinase activity (Supplementary Figure S1A). From our dataset of ∼17,000 phosphopeptides (Supplementary Data S2), we identified >800 significantly upregulated and >1,000 significantly downregulated phosphopeptides across both ONC201 and TR-57 treatments in WT SUM159 cells. Between both treatments, 276 upregulated and 478 downregulated phosphopeptides were observed (Supplementary Figure S1B). Conversely, only 54 significantly upregulated phosphopeptides and 109 significantly downregulated phosphopeptides were found in ClpP-KO SUM159 cells across both treatments.

Using RoKAI (Yılmaz et al., 2021) to identify patterns of kinase activity from our phosphoproteomics data, we found evidence for activation of multiple kinases in WT SUM159 cells treated with either ONC201 or TR-57 (Supplementary Figure S1C), including ATM, ATR, AMPK, CK2, MAPKAPK2 and others. Similarly, RoKAI predicted the kinases CDK2, CDK4, and AurB to be inactivated under these same conditions. Many of these predictions were determined to be ClpP-dependent, as ClpP-KO cells treated with ONC201 or TR-57 showed few predicted changes in kinase activity following treatment (Supplementary Figure S1C).

#### Transcriptomics

RNAseq was performed on mRNA isolated from both WT and ClpP-KO cells following a 24-h treatment with either 0.1% DMSO, 10 μM ONC201, or 150 nM TR-57. Volcano plots of the processed data showed significant changes in the WT cells following ONC201 and TR-57 treatment (Figure 2C). Specifically, >700 and >1,000 transcripts were significantly altered in ONC201 or TR-57 treated WT cells, respectively. Of these, 501 upregulated transcripts were identical between both treatments, whereas 492 downregulated transcripts were common (Figure 2D). Again, ClpP-KO cells showed greatly reduced statistically significant changes, with <220 upregulated and <130 downregulated transcripts following 24-h treatment. To investigate short-term effects of ClpP on the transcriptome, we performed transcriptomic analysis following 1 h of ONC201 or TR-57 treatment in WT and ClpP-KO SUM159 cells (Supplementary Figure S3). In these early timepoints, we observed significant upregulation of 80 and 115 genes in WT cells treated with ONC201 and TR-57 respectively, as well as significant downregulation of 174 and 200 genes in these same samples. ClpP-KO SUM159 showed significant changes in 83 upregulated and 94 downregulated genes following ONC201 treatment, and 115 upregulated and 113 downregulated genes in TR-57 treated cells. Significant overlap of changes occurring in WT cells treated with both drugs was seen; however, significant overlap was not observed between WT and ClpP-KO treated cells (Supplementary Figures S3B,C). GO analysis of 1 h transcript changes reveals significant downregulation of cytosolic transcripts in both ONC201 and TR-57 treated WT cells. Additionally, significant upregulation of cytosolic and nucleoplasmic transcripts was observed in ONC201 and TR-57 treated WT cells, respectively (Supplementary Figure S3D). Comparison of the 1 h and 24 h treatments with either ONC201 or TR-57 in WT cells did not reveal significant overlap of transcriptional changes (data not shown). Further investigation into transcriptomic changes following ClpP activation with additional timepoints is necessary to better characterize the temporal regulation of transcription following ClpP activation in TNBC cells.

GO analysis of the significantly up- and downregulated transcripts from WT cells revealed that many these transcripts are nuclear or cytosolic. In contrast to our proteomics results, the most significantly downregulated transcripts were those encoding proteins localized to the cytoplasm, cell membrane, nucleus and other non-mitochondrial organelles (Supplementary Figures S2C,D). Additionally, these same subcellular localizations were significantly enriched in the analysis of upregulated transcripts (Supplementary Figure S2C), including transcripts for FLRT1, SKIL, SLC25A6, SLC6A9, and ASNS (Supplementary Figure S2D). Transcriptomic results also showed significant downregulation of some nuclear-encoded transcripts for mitochondrial proteins; however, this was not observed to the same extent as the proteomics data (Supplementary Figure S2A,C).

#### Metabolomics

Individual analysis of the metabolomics data (specifically, ontology levels OL1, OL2a/b, PDa; see Materials and Methods) showed significant ClpP-dependent downregulation of specific metabolites related to mitochondrial function (Figure 2E). Metabolomics data from WT cells showed a high degree of overlap between ONC201 and TR-57 treatment conditions, with 30 upregulated metabolites and 47 downregulated metabolites consistent across conditions (Figure 2F). We observed decreases in ascorbic acid, propionylcarnitine, and ureidosuccinic acid (Supplementary Figure S2E), metabolites which are involved in redox balance, acylcarnitine metabolism, and aspartic acid catabolism, respectively. Our metabolomics analysis also showed a significant upregulation in α-ketoglutarate and 2-hydroxyglutaric acid, two key metabolites linked to TCA cycle dysfunction and epigenetics regulation (Losman and Kaelin, 2013; Vander Heiden and Deberardinis, 2017). Significant changes in multiple di- and tripeptides was also observed (Supplementary Data S6).

### Multi-omics comparative analysis reveals significantly altered mitochondrial metabolism in TNBC cells

**FIGURE 3:**
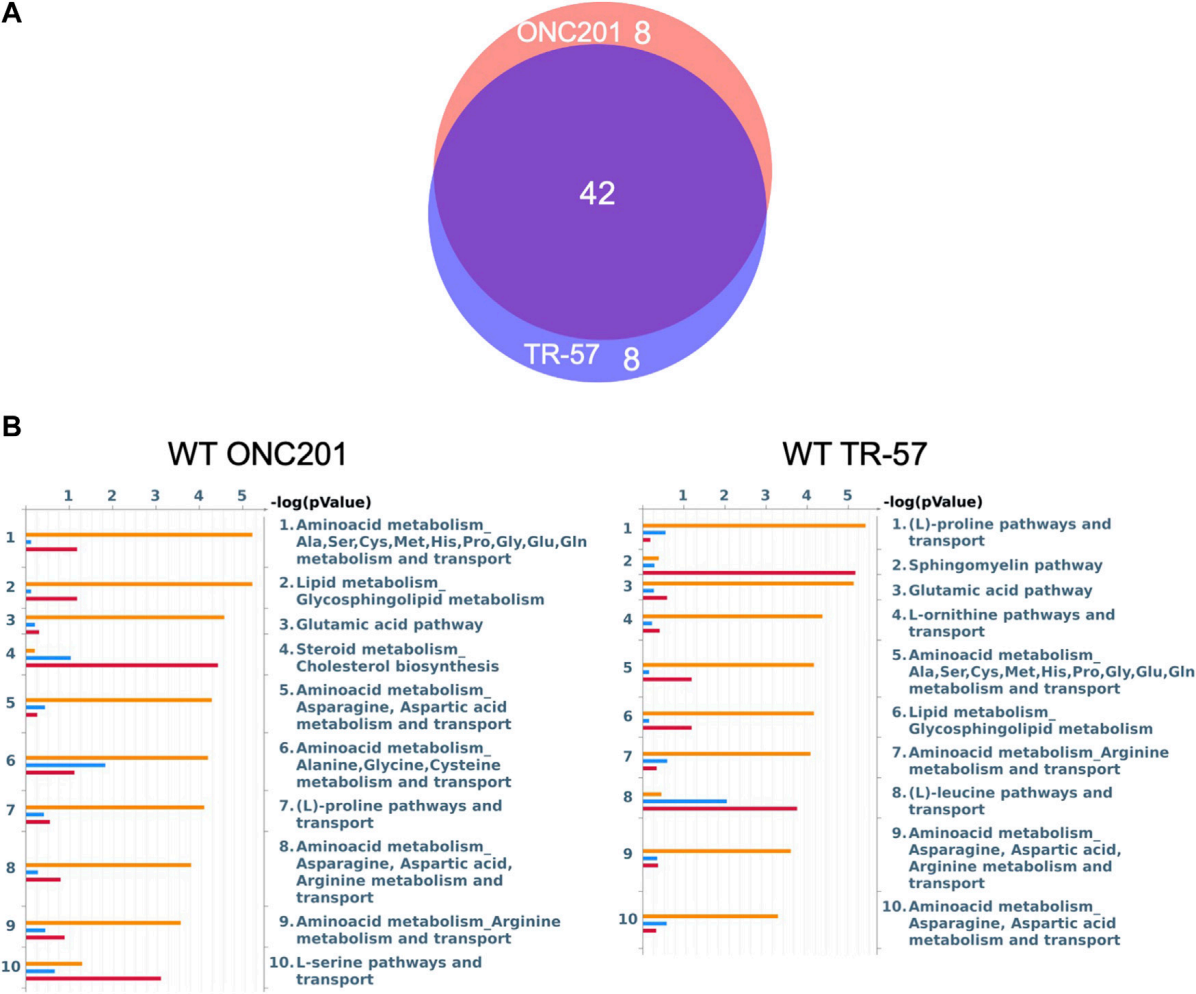
MetaCore pathway analysis reveals multiple pathways affected at the proteomic, transcriptomic, and metabolomic level with potential for further investigation in triple-negative breast cancer cells. **(A)** Venn diagram endogenous metabolic network (EMN) overlap between ONC201 and TR-57 treated WT SUM159 cells. Values represent number of pathways found in each group using data from Figure 2. **(B)** MetaCore Endogenous Metabolic Network (EMN) analysis from proteomic (blue), metabolomic (orange), and transcriptomic (red) datasets for WT SUM159 cells treated with 10 μM ONC201 or 150 nM TR-57.

To further evaluate the effects of ClpP activation on TNBC cells, MetaCore pathway analysis software was used to perform multi-omics analysis of all the data from ONC201 and TR-57 treated WT and ClpP-KO SUM159 cells. Each cell line and treatment group were analyzed in comparison to their respective DMSO control, and observations meeting significance criteria from all three datasets (proteomics, transcriptomics, and metabolomics) were used for each analysis when available. From this analysis, the top 50 significantly altered endogenous metabolic networks (EMNs) were identified by MetaCore software, with 42 of these 50 EMNs occurring in both ONC201 and TR-57 treated WT SUM159 cells (Figure 3). From this analysis we detected significant perturbations in pathways involving amino acid metabolism and transport, TCA cycle, glycosphingolipid, citrulline, and sucrose metabolism (Table 1). To determine ClpP-dependence of these EMN perturbations, we performed the same MetaCore analysis for ClpP-KO cells treated with ONC201 or TR-57. This yielded no significant EMNs for ONC201 treated ClpP-KO SUM159 cells. Additionally, proteomics observations meeting the significance criteria for TR-57 treatment did not occur in ClpP-KO SUM159 cells. For these reasons, MetaCore analysis of ClpP-KO SUM159 cells was performed using only transcriptomic and metabolomic datasets (Supplementary Figure S4, Supplementary Table S1).

**TABLE 1:** ClpP activation perturbs many metabolic pathways across proteomic, transcriptomic, and metabolomic landscapes in triple-negative breast cancer cells. Representative top endogenous metabolic networks (EMN) identified using MetaCore for each group, as outlined in Figure 3. Number of hits (perturbed EMN members) and *p*-value are listed for each dataset in the order they appear in the group name.

**Group**	**Metabolic networks**	**Hits**	*p* **-Value**
ONC201	*1-acyl-glycerol, 3-phosphocholine pathway*	6/88	1.08 × 10^−1^
	*Glycine pathway*	10/90	1.14 × 10^−1^
	*1-oleoyl-sn-glycerol-3-phosphocholine pathway*	8/121	3.56 × 10^−1^
TR-57	*Sphingomyelin pathway*	17/97	6.77 × 10^−6^
	*Carbohydrate metabolism, Glycolysis, Glucogenesis, and glucose transport*	7/134	4.00 × 10^−1^
	*D-glucuronic acid pathway*	10/77	9.53 × 10^−3^
ONC201 and TR-57	*Amino acid metabolism: Ala, Ser, Cys, Met, His, Pro, Gly, Glu, Gln metabolism and transport*	26, 24/190	5.92 × 10^−6^, 7.14 × 10^−5^
	*Glutamic acid pathway*	14, 16/103	2.72 × 10^−5^, 7.47 × 10^−6^
	*Tyrosine pathway*	11, 14/90	2.69 × 10^−3^, 7.57 × 10^-4^
	*Lipid Metabolism, Glycosphingolipid metabolism*	26, 24/190	5.92 × 10^−6^, 7.14 × 10^−5^
	*Amino acid metabolism: Asn, Asp metabolism and transport*	15, 14/112	5.33 × 10^−5^, 5.58 × 10^-4^
	*Amino acid metabolism: Ala, Gly, Cys metabolism and transport*	22, 23/115	6.56 × 10^−5^, 6.66 × 10^-4^
	*Steroid metabolism, Cholesterol biosynthesis*	17, 14/88	3.84 × 10^−5^, 1.05 × 10^−3^
	*Vitamin, mediator, and cofactor metabolism: CoA biosynthesis and transport*	12, 7/81	3.77 × 10^-2^, 1.59 × 10^−1^
	*Sucrose pathway*	9, 11/90	2.92 × 10^−1^, 1.44 × 10^-2^
	*(L)-leucine pathways and transport*	13, 17/85	1.96 × 10^-2^, 1.87 × 10^-4^
	*(L)-proline pathways and transport*	17, 16/118	8.04 × 10^−5^, 3.75 × 10^−6^
	*(S)-citrulline pathway*	12, 10/75	9.65 × 10^−3^, 5.04 × 10^-2^
	*Carbohydrate metabolism: TCA and tricarboxylic acid transport*	15, 13/101	9.38 × 10^-4^, 1.50 × 10^−3^

Italic values are the names of the metabolic networks.

#### TCA cycle

**FIGURE 4:**
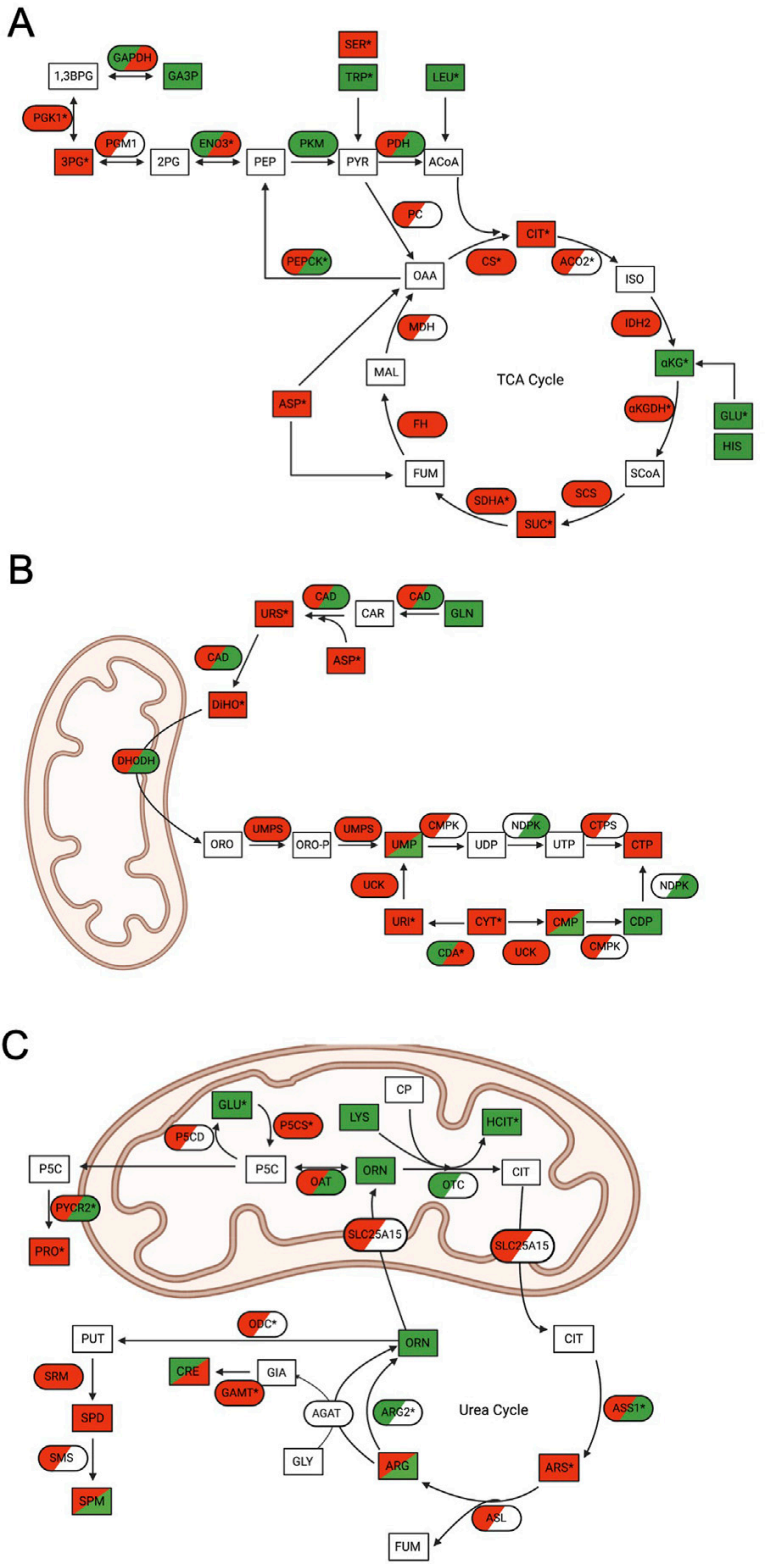
Multi-omics analysis reveals significant mitochondrial metabolic pathway perturbations following ONC201 or TR-57 treatment in SUM159 cells. In all schematics, rectangles represent metabolites and ovals represent enzymes. Directionality of change is indicated by color (downregulation (red), upregulation (green), not detected (white)) and * denotes significant change in at least one dataset. Dual-colored enzymes represent those with different directional changes in proteomics (left side) and transcriptomics (right side) while dual-colored metabolites indicate different directional changes in ONC201 treatment (left side) and TR-57 treatment (right side). **(A)** Schematic of the tricarboxylic acid (TCA) cycle. CIT: *citrate*; ISO: *isocitrate*; αKG: *α-ketoglutarate*; GLU; *glutamic acid*; HIS: *histidine*; SCoA: *succinyl-CoA*; SUC: *succinate*; FUM: *fumarate*; MAL: *malate*: OAA: *oxaloacetate*; ASP: *aspartic acid*; ACoA: *acetyl-CoA*; PYR: *pyruvate*; PEP: *phosphoenolpyruvate*: TRP: *tryptophan*; SER: *serine*; LEU: *leucine*; 2 PG: *2-phosphoglycerate*; 3 PG: *3-phosphoglycerate*; 1,3BPG: *1,3-bisphosphoglycerate*; GA3P: *glyceraldehyde-3-phosphate*; ACO2: *aconitase 2*; IDH2: *isocitrate dehydrogenase 2*; αKGDH: *α-ketoglutarate dehydrogenase*; SCS: *succinyl-CoA synthetase*; SDHA: *succinate dehydrogenase A*; FH: *fumarase*: MDH: *malate dehydrogenase*; PC: *pyruvate carboxylase*; PEPCK: *phosphoenolpyruvate carboxykinase*; PDH: *pyruvate dehydrogenase*; PKM: *pyruvate kinase*; ENO3: *β-enolase*; PGM1: *phosphoglycerate mutase 1*; PGK1: *phosphoglycerate kinase 1;* GAPDH: *glyceraldehyde phosphate dehydrogenase*. **(B)** Schematic of *de novo* and scavenging pathways of pyrimidine synthesis. GLN: *glutamine*; ASP: *aspartic acid*; URS: *ureidosuccinic acid*; DiHO: *dihydroorotate*; ORO: *orotate*; ORO-P: *phospho-orotate*; UMP: *uridine 5′-monophosphate*; UDP: *uridine 5′-diphosphate*; UTP: *uridine 5′-triphosphate*; CMP: *cytidine 5′-monophosphate*; CDP: *cytidine 5′-diphosphate*; CTP: *cytidine 5′-triphosphate*; URI: *uridine*; CYT: *cytidine*; CAD: *carbamoyl-phosphate synthetase 2, aspartate trasncarbamylase, and dihydroorotase*; DHODH: *dihydroorotate dehydrogenase*; UMPS: *uridine 5′-monophosphate synthase*; CMPK: *UMP-CMP kinase*; NDPK: *nucleoside diphosphate kinase*; CTPS: *CTP synthase*; UCK: *uridine-cytidine kinase*; CDA: *cytidine deaminase*. **(C)** Schematic of urea cycle and citrulline pathway. CIT: *citrulline*; ARS: *argininosuccinate*; FUM: *fumarate*; ARG: *arginine*; ORN: *ornithine*; CP: *carbamoyl phosphate*; LYS: *lysine*; HCIT: *homocitrulline*; GLY: *glycine*; GIA: *guanidinoacetate*; CRE: *creatine*; PUT: *putrescine*; SPD: *spermidine*; SPM: *spermine*; P5C: *1-pyrroline-5-carboxylic acid*; PRO: *proline*; GLU: *glutamic acid*; OTC: *ornithine transcarbamylase*; SLC25A15: *mitochondrial ornithine transporter 1*; ASS1: *argininosuccinate synthase*; ASL: *argininosuccinate lyase*; ARG2: *arginase-2*; ODC: *ornithine decarboxylase*; SRM: *spermidine synthase*; SMS: *spermine synthase*; AGAT: *glycine amidinotransferase*; GAMT: *guanidinoacetate N-methyltransferase*; OAT: *ornithine aminotransferase*: P5CS: *1-pyrroline-5-carboxylic acid synthase*; P5CD: *1-pyrroline-5-carboxylic acid dehydrogenase*; PYCR2: *Pyrroline-5-carboxylate reductase 2*.

Within these pathways, multiple proteins, transcripts, and metabolites were identified as significantly up- or downregulated in WT cells through MetaCore EMN analysis (Figure 3). Analysis of these pathway changes showed that significant perturbations in all three datasets contributed to their enrichment (Figure 4). Within the TCA cycle, proteomic downregulation was observed for citrate synthase (CS), isocitrate dehydrogenase 2 (IDH2), α-ketoglutarate dehydrogenase (αKGDH), succinyl-CoA synthetase (CSC), succinate dehydrogenase A (SDHA), fumarate hydratase (FH), and malate dehydrogenase (MDH) (Figure 4A), consistent with previous observations by immunoblot (Fennell et al., 2022) and BioID (Ishizawa et al., 2019). Significant proteomic downregulation of aconitase (ACO2) was observed in both ONC201 and TR-57 treated WT SUM159 cells (Figure 4A). Additionally, transcriptomic downregulation was observed for many of these TCA cycle enzymes, including significant downregulation of CS in TR-57 treated cells and αKGDH and SDHA in ONC201 treated cells (Figure 4A). Suggesting potential compensation towards glycolysis, β-enolase (ENO3) showed significant proteomic upregulation following both ONC201 and TR-57 treatment. Similarly, phosphoenolpyruvate carboxykinase (PEPCK) showed significant transcriptomic upregulation following both treatments. Metabolites including citrate, succinate, and 3-phosphoglycerate were significantly decreased following both ONC201 and TR-57 treatment in WT cells while α-ketoglutarate was significantly upregulated. Glucogenic amino acids tryptophan and glutamic acid were both significantly upregulated while serine was significantly downregulated. The ketogenic amino acid leucine was significantly upregulated (Figure 4A). While also identified as a significant EMN for TR-57 treated ClpP-KO SUM159 cells, this was on the basis of one transcript and one metabolite being present in the data (Supplementary Figure S4, Supplementary Data S7).

#### Pyrimidine metabolism

Our data analysis identified that the *de novo* pyrimidine synthesis and scavenging pathway, a cytoplasmic and mitochondrial metabolic pathway, was significantly downregulated (Figure 4B). We observed the trend of proteomic downregulation amongst most enzymes involved in this pathway and significant transcriptomic downregulation of cytidine deaminase (CDA). Substrates and metabolites of *de novo* pyrimidine biosynthesis, including aspartic acid, ureidosuccinic acid, and dihydroorotate, were downregulated as well as uridine and cytidine.

#### Citrulline pathway

The (S)-citrulline pathway/urea cycle, another mitochondrial and cytosolic pathway, showed many significant changes following ONC201 or TR-57 treatment (Figure 4C). A consistent protein-level downregulation was observed with delta-1-pyrroline-5-carboxylate synthase (P5CS) and ornithine decarboxylase (ODC) following TR-57 and ONC201 treatment, respectively. P5CS and ODC are part of the glutamine catabolic pathway leading to citrulline biosynthesis. Moreover, pyrroline-5-carboxylate reductase (PYCR2), part of the proline biosynthesis pathway was strongly downregulated consistent with a previous report in TNBC cells (Greer et al., 2022). Mitochondrial arginase 2 (ARG2) was significantly increased at the proteomic level following both compound treatments. In comparison, upregulation of PYCR2 and argininosuccinate synthetase (ASS1) was observed at the transcriptomic level, whereas significant transcriptomic downregulation was also observed with guanidinoacetate N-methyltransferase (GAMT) and P5CS following ONC201 treatment. Glutamic acid and homocitrulline were both significantly upregulated in the metabolomic data following compound treatment, while argininosuccinic acid (ARS) was significantly decreased. S-adenosylmethionine, the methyl-donor in the reaction catalyzed by GAMT, was also significantly upregulated following both compound treatments. The S-citrulline pathway was also identified as significant in the EMN analysis of ClpP-KO SUM159 cells treated with TR-57. However, similar to the TCA cycle, this was due to the presence of one metabolite and three transcripts (Supplementary Figure S4, Supplementary Data S7).

##### ClpP activation induces ATF4 expression and affects ATF4-dependent gene expression

**FIGURE 5:**
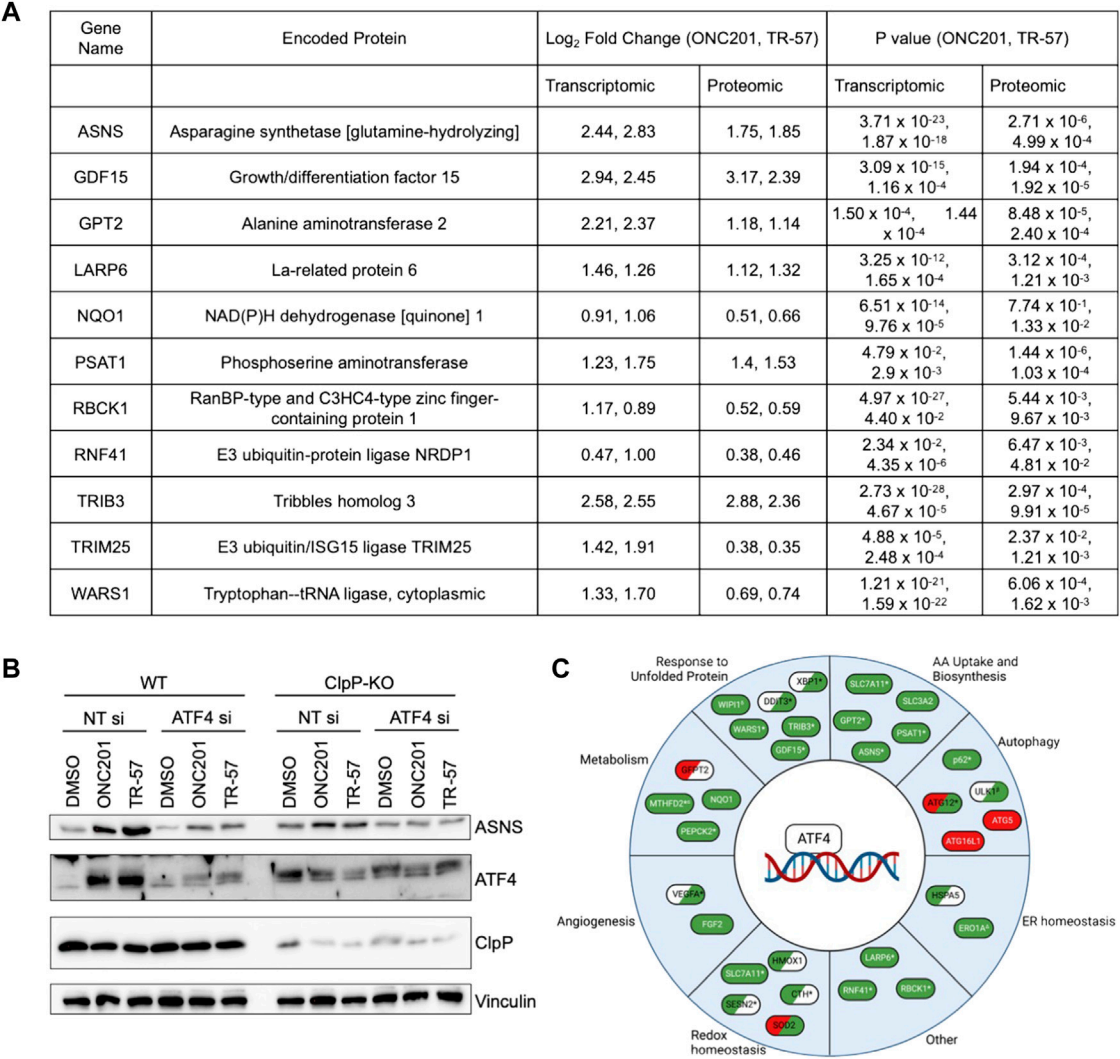
Pharmacological ClpP activation leads to ATF4 induction and increase in ATF4 target genes in TNBC cells. **(A)** Table of selected ATF4-dependent genes/proteins and their measured Log_2_(Fold Change) and *p*-value from proteomics and transcriptomics data from WT SUM159 cells treated with 10 μM ONC201 and 150 nM TR-57 for 24 h. **(B)** Immunoblot of SUM159 cells treated with 0.1% DMSO, 10 μM ONC201, or 150 nM TR-57 for 24 h. Cells were either treated with a non-targeting siRNA or an ATF4 siRNA. Representative of N = 2. **(C)** Schematic of ATF4 target genes and their involvement in diverse cellular processes. Green: transcripts and protein were upregulated in WT SUM159 cells treated with either ONC201 or TR-57. Red: transcripts and protein were downregulated in WT SUM159 cells treated with either ONC201 or TR-57. White: not detected in our datasets. Items in two colors indicate direction of change in proteomics (left) and transcriptomics (right). *At least one dataset is significant (*p*-value < 0.05); ^α^TR-57 treated SUM159 cells showed decreased proteomic values; ^β^TR-57 treated SUM159 cells showed decreased transcriptomic values; ^Δ^TR-57 treated SUM159 cells showed decreased proteomic and transcriptomic values; ^δ^ Not detected in TR-57 treated SUM159 cells (proteomics).

MetaCore pathway analysis revealed significant changes in amino acid metabolism following pharmacological ClpP activation (Figure 3B; Table 1). EMN analysis showed significant perturbations in proteins and transcripts including ASNS, SHMT2, GPT2, and phosphoserine aminotransferase (PSAT1) (Supplementary Data S7), genes whose expression have been previously reported to be ATF4-dependent (Su and Kilberg, 2008; Wang et al., 2015; Quirós et al., 2017). Further investigation of the proteomic and transcriptomic data revealed that many ATF4-dependent genes were significantly upregulated at both the transcript- and protein-level following activation of ClpP with ONC201 or TR-57 (Figure 5A). Consistent with these data, the induction of ATF4 and ASNS was validated at the protein level by immunoblotting (Figure 5B). ASNS protein expression was induced in WT SUM159 but not ClpP-KO SUM159 cells following 24-h treatment with either 10 μM ONC201 or 150 nM TR-57. Additionally, increases in ASNS were not observed in WT SUM159 cells treated with an ATF4 siRNA prior to ONC201 or TR-57 incubation, indicating that expression of these proteins was ATF4-dependent (Figure 5B). ATF4 regulates a number of processes, and further investigation into potential additional ATF4 substrates (Wang et al., 2015) identified multiple significantly upregulated enzymes involved in metabolic processes (e.g., PEPCK2), autophagy (e.g., p62), and redox homeostasis (e.g., HMOX) (Figure 5C).

##### MetaCore pathway analysis identifies multiple amino acid metabolic pathways affected by ONC201 or TR-57

MetaCore pathway analysis identified amino acid metabolism, including serine metabolism, as significantly affected by ONC201 and TR-57 treatment (Table 1). Significant upregulation of PSAT1 (Figures 5A,C) through ATF4 indicates a potential upregulation of the serine biosynthetic pathway (Supplementary Figure S5A). We observed significant upregulation of phosphoglycerate dehydrogenase (PHGDH), and PSAT1 at both the proteomic and transcriptomic level and phosphoserine phosphatase (PSPH) at the proteomic level. Additionally, we observed significant loss of 3-phosphoglycerate and serine following ONC201 and TR-57 treatment. Recently, Pacold et al. identified a novel PHGDH inhibitor, NCT-503 (Pacold et al., 2016), and demonstrated its ability to inhibit cell growth in TNBC cells highly expressing PHGDH, but not in TNBC cells expressing low levels of PHGDH. Because ONC201 and TR-57 induce PHGDH expression in SUM159 cells, we investigated whether NCT-503 would further sensitize SUM159 cells to TR-57 or *vice versa*. Despite using relatively high levels of NCT-503 (up to 50 μM), SUM159 cells were not inherently sensitive to NCT-503 (Supplementary Figure S5B). Additionally, treatment of SUM159 cells with 150 nM TR-57 did not alter the IC_50_ of cells to NCT-503, nor did 10 μM NCT-503 affect the IC_50_ of cells to TR-57 (Supplementary Figure S5B).

##### ONC201 and TR-57 affect the heme biosynthetic pathway in TNBC cells

**FIGURE 6:**
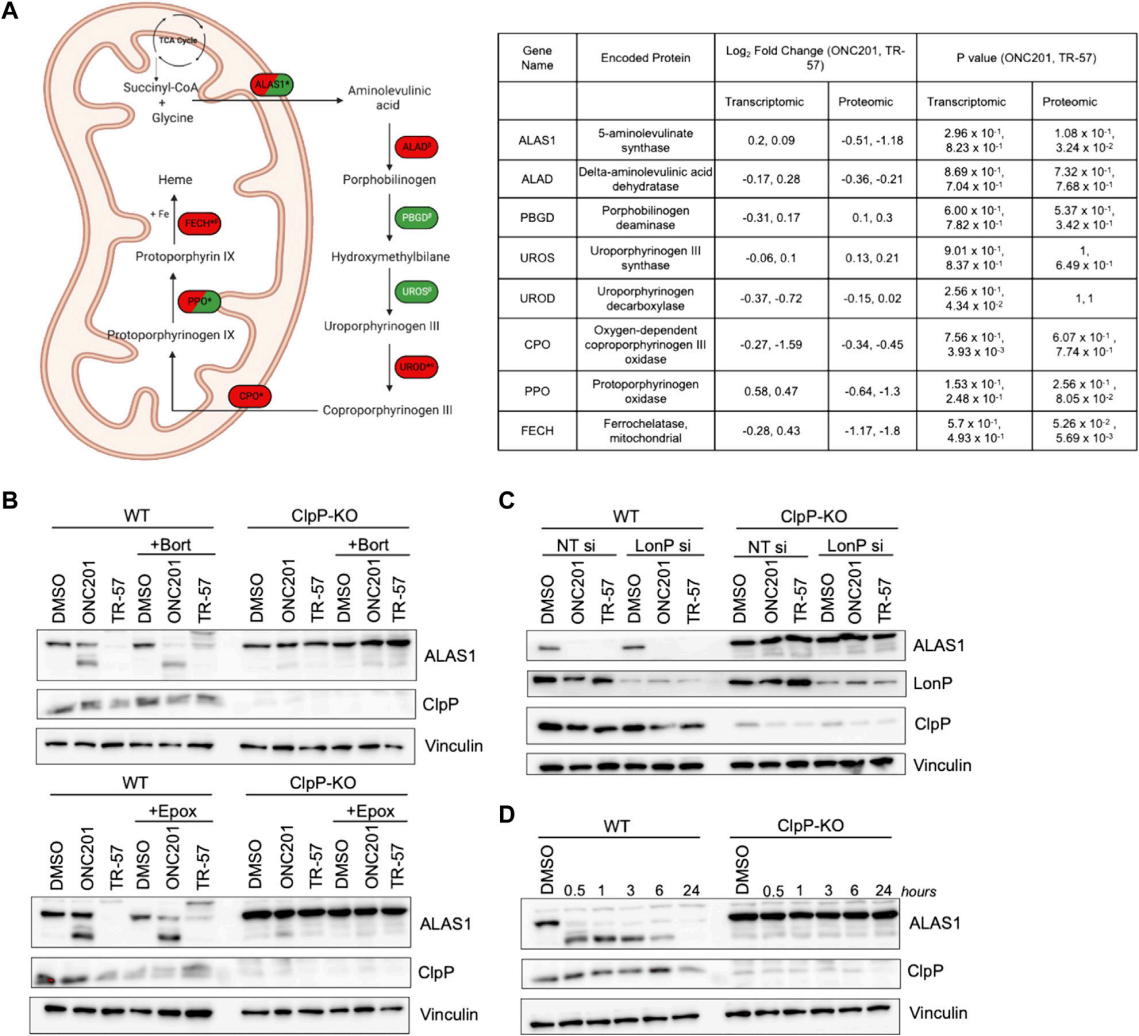
ClpP activation induces loss of major components of the heme biosynthetic pathway in TNBC cells. **(A)** Schematic of heme biosynthetic pathway (left). Directionality of change is indicated by color (downregulation (red), upregulation (green), not detected (white)). * indicates significant in at least one dataset, conflicting ^α^proteomic, ^β^transcriptomic, or ^Δ^both datasets for one treatment. ^δ^Not detected in TR-57 proteomics dataset. Dual-colored enzymes represent those with different directional changes in proteomics (left side) and transcriptomics (right side) while dual-colored metabolites indicate different directional changes in ONC201 treatment (left side) and TR-57 treatment (right side). ALAS1: 5′-*aminolevulinate synthase 1*; ALAD: *aminolevulinate dehydratase*; PBGD: *porphobilinogen deaminase*; UROS: *uroporphyrinogen III synthase*; UROD: *uroporphyrinogen decarboxylase*; CPO: *coproporphyrinogen oxidase*; PPO: *protoporphyrinogen oxidase*; FECH: *ferrochelatase*. WT and ClpP-KO SUM159 cells were immunoblotted for ALAS1 following 24 h of 10 μM ONC201 or 150 nM TR-57 fin the presence or absence of **(B)** 10 nM bortezomib (Bort) or 1 μM epoxomicin (Epox) or **(C)** LonP siRNA knockdown (LonP si). Cells were reverse transfected with either non-targeting or LonP1-targeting siRNA for 48 h before ClpP activator treatment. **(D)** Time-dependent immunoblot of 150 nM TR-57 treatment in WT and ClpP-KO SUM159 cells. All blots are representative of N = 2.

MetaCore pathway analysis also revealed significant changes in glycine pathways and cellular transport following ClpP activation. Glycine and succinyl-CoA are substrates required for heme biosynthesis through the rate-limiting enzyme ALAS1. Our proteomics data revealed that ALAS1, PPO, and FECH, three key enzymes required for heme biosynthesis, were downregulated following ONC201 and TR-57 treatment. By contrast, the transcript levels of ALAS1, PPO, and FECH, were not significantly changed following ONC201 or TR-57 treatment, suggesting that loss of these proteins is due to degradation caused by ClpP activation (Figure 6A). Immunoblot analysis validated ONC201 and TR-57 mediated ALAS1 decline in WT, but not in ClpP KO SUM159 cells (Figure 6B), consistent with previous reports of ClpP degrading ALAS1 (Kubota et al., 2016; Wong and Houry, 2019; Yien and Perfetto, 2022). The downregulation of ALAS1 occurred as early as 30 min of treatment with TR-57 in WT, but not knockout SUM159 cells (Figure 6D).

Since LonP, another mitochondrial matrix protease, has also been reported to degrade ALAS1 (Tian et al., 2011; Kubota et al., 2016; Yien and Perfetto, 2022), we investigated whether the loss of ALAS1 was due to ClpP or LonP activation. To accomplish this, bortezomib (10 nM) was used to inhibit both LonP and the proteasome, whereas epoxomicin (1 μM) was used as an inhibitor of only the proteasome (Lu et al., 2013; Csizmadia et al., 2016; Lee et al., 2022). Neither of these co-treatments with ONC201 or TR-57 prevented the loss of ALAS1 (Figure 6B). LonP siRNA was also used to examine whether LonP was responsible for the observed loss of ALAS1 in WT SUM159 cells (Figure 6C). Transient knockdown of LonP did not prevent the degradation of ALAS1 in WT SUM159 cells following treatment, indicating that the effect of ONC201 or TR-57 on ALAS1 was due to activation of ClpP.

Because our data identified components of the heme metabolic pathway as being significantly downregulated by ONC201 and TR-57 treatment at the protein level, we next investigated whether inhibition of heme biosynthesis was required for growth arrest in response to these compounds. To accomplish this, we supplemented the cell media with hemin, a cell-permeable form of heme and examined the effects on cell growth inhibition following ONC201 or TR-57 treatment. Cell proliferation assays (72-h) did not show restoration of cell growth following hemin supplementation (Supplementary Figure S6A). However, to further determine if hemin supplementation over an extended exposure would prevent growth inhibition, we used long-term hemin exposure in crystal violet assays (Supplementary Figure S6B, Supplementary Figure S7). These results demonstrated that extended hemin supplementation did not counteract cell growth inhibition following ONC201 or TR-57 treatment in either WT SUM159 or MDA-MB-231 cells.

## Discussion

The mechanism of action of ONC201 has been proposed as dopamine receptor antagonism (Allen et al., 2016; Arrillaga-Romany et al., 2017; Kline et al., 2018), atypical activation of the ISR (Allen et al., 2016; Ishizawa et al., 2016; Kline et al., 2016), and more recently, ClpP agonism (Graves et al., 2019; Ishizawa et al., 2019). We and others have shown that ONC201 and the related TR compounds affect mitochondrial metabolic function (e.g., OXPHOS, TCA cycle) and mitochondrial protein levels in cancer models (Greer et al., 2018; Ishizawa et al., 2019; Fennell et al., 2022; Greer et al., 2022; Nguyen et al., 2022). The current study utilized a comprehensive multi-omics approach to identify protein, transcript, and metabolite-level changes to further elucidate the specific mechanism of action of these compounds and identify potential cancer cell vulnerabilities resulting from ClpP activation.

We identified many protein, transcript, and metabolite perturbations occurring in TNBC cells following a brief (24-h) exposure to ONC201 or TR-57. These perturbations were determined to be largely ClpP dependent as most were not observed in ClpP-KO cells (Figure 2). We previously showed ONC201 and TR compound treatment result in similar phenotypic responses in TNBC cells (Fennell et al., 2022; Greer et al., 2022) and comparisons of the responses to these structurally distinct ClpP activators in WT SUM159 cells demonstrated highly similar responses to these compounds. Thus, our data further illustrates the high degree of specificity of these compounds for ClpP and confirm that this is the major target protein for these molecules in cancer cells.

GO analysis of significantly downregulated proteins revealed multiple mitochondrial proteins, including mitochondrial matrix and inner membrane proteins, in both treatments. Mitochondrial translation and mitochondrial protein transport were identified as significantly downregulated at the proteomic level (Supplementary Figure S2A). Significant decreases in nuclear-encoded mitochondrial transcripts were also identified (Supplementary Figure S2C), however, the number of identified transcripts does not fully account for the overall observed decline in mitochondrial proteins, supporting the loss of mitochondrial protein as due to direct proteolysis. In agreement with activation of the ISR or UPR^mt^, upregulated proteins and transcripts showed a strong enrichment for nuclear and cytosolic components, as well as non-mitochondrial organelles (e.g., endoplasmic reticulum and Golgi apparatus) and membrane components, as a likely compensatory response to this stress.

Multi-omics analysis using MetaCore pathway analysis identified multiple metabolic networks that were significantly altered by ClpP activation in WT SUM159 cells, including amino acid biosynthesis and the (S)-citrulline pathway (Table 1; Figure 3, Figure 4). We also found that the TCA cycle was significantly downregulated at both the proteomic and transcriptomic level following ClpP activation. Additionally, we observed significant downregulation of TCA cycle intermediates citrate and succinate, and upregulation of α-ketoglutarate (Figure 4A). Loss of succinate dehydrogenase (SDH), isocitrate dehydrogenase (IDH2), and ACO2 were previously reported following treatment with ONC201 or TR compounds (Ishizawa et al., 2019; Fennell et al., 2022). The TCA cycle serves as a source for both anabolic metabolism intermediates as well as NADH and TR-57 has been reported to reduce NAD^+^ and NADH in a ClpP-dependent manner and induce redox imbalance in TNBC cells (Greer et al., 2022). Decreased flux through the TCA cycle, as well as loss of SDH (complex II of the electron transport chain), could explain the observed loss of OXPHOS following ClpP activation (Greer et al., 2018; Fennell et al., 2022; Nguyen et al., 2022).

The citrulline pathway, including the urea cycle, was identified as a significantly perturbed pathway from our multi-omics analysis (Figure 4C). We observed downregulation of arginosuccinate synthase 1 (ASS1) on a proteomic level, which is proposed to preserve aspartate levels in cancer cells for use in biosynthetic pathways. This proteomic decline was accompanied by a significant transcriptomic upregulation of ASS1 in WT cells following both ONC201 and TR-57 treatment. We also observed significant proteomic upregulation of arginase (ARG2) following ClpP activation. Additionally, we observed a significant decrease in both aspartate and argininosuccinate (Figure 4C), which may be due to the significant upregulation of ASNS (Figure 5), which depletes aspartate through conversion to asparagine. Loss of arginine synthesis was reported to suppress transcription of genes including those involved in OXPHOS, glycolysis, and nucleotide biosynthesis, and alterations in mitochondrial morphology (Qiu et al., 2015; Cheng et al., 2018; Chen et al., 2021), potentially explaining some phenotypes previously observed following ClpP activation (Greer et al., 2018; Fennell et al., 2022; Greer et al., 2022).

Aspartate is also required for *de novo* pyrimidine biosynthesis, and further analysis of *de novo* and scavenging pathways of pyrimidine biosynthesis showed significant downregulation of metabolites including aspartate, dihydroorotate, uridine, and cytosine. We observed a proteomic downregulation of CAD protein, a trifunctional enzyme facilitating the conversion of carbamoyl-phosphate to dihydroorotate. Interestingly, the key mitochondrial enzyme (DHODH) and 4th enzyme in the pyrimidine biosynthetic pathway, was not reduced in our proteomics analysis. Inhibition of *de novo* nucleotide biosynthesis has been widely investigated as an anticancer therapy and as a metabolic vulnerability to be exploited in co-treatment development (Huang and Graves, 2003; Luengo et al., 2017). As pyrimidine nucleotides are required for continued cell proliferation, the observed downregulation of pyrimidine biosynthetic metabolites and enzymes could play a critical role in the mechanism by which ClpP activation leads to cell growth inhibition.

Dependence on serine biosynthesis was previously identified as a metabolic vulnerability in cancer models, including breast cancer (Luengo et al., 2017). We identified significantly decreased serine levels with significant proteomic and transcriptomic upregulation of the serine biosynthetic pathway in cells treated with ONC201 or TR-57 (Table 1, Supplementary Figure 6S5A). This is indicative of cells potentially upregulating this pathway to synthesize more serine for utilization by other pathways (e.g., tetrahydrofolate cycle, glycine synthesis, glutathione synthesis). Inhibition of PHGDH was reported to inhibit cell growth in cells highly expressing PHGDH (Pacold et al., 2016). We hypothesized that if dependence on serine biosynthesis is a metabolic vulnerability in cells treated with ClpP activators, that a combination treatment with NCT-503, a novel PHGDH inhibitor (Pacold et al., 2016), would sensitize cells to ClpP activators. We did not observe sensitization of WT SUM159 cells to TR-57, nor did TR-57 co-treatment sensitize cells to NCT-503. However, despite induction of PHGDH following TR-57 treatment, SUM159 may not express PHGDH to the extent required for cells to be sensitive to NCT-503 treatment alone (Pacold et al., 2016). Importantly, while our metabolomics data provided a snapshot of the metabolome following 24-h treatment with ClpP agonists it cannot not reflect the actual flux through the serine biosynthetic pathway or the use of serine in other metabolic pathways, or the uptake of serine from cell growth media. Future studies involving more detailed analysis of metabolic flux using stable isotope approaches, could be applied to investigate differences in pathway flux.

Consistent with perturbations in amino acid biosynthesis, analysis of significantly upregulated proteins and transcripts identified many ATF4-dependent genes, including ASNS, GDF15, and PSAT1 (Figure 5). ATF4 has been widely shown to be induced by ClpP activation (Kline et al., 2016; Wagner et al., 2017; Yuan et al., 2017; Graves et al., 2019). ATF4, a key regulator of the cellular response to the ISR/UPR^mt^, is known to regulate transcription of genes involved in amino acid uptake and biosynthesis, metabolic enzymes, and redox homeostasis (Wang et al., 2015; Quirós et al., 2017). We identified upregulation of ASNS following ClpP activation in both proteomic and transcriptomic data, and this was confirmed by immunoblotting (Figure 5). Additionally, we showed that the upregulation of ASNS was prevented in ATF4-knockdown SUM159 cells treated with ONC201 and TR-57. The observed activation of ATF4 and ATF4-dependent genes may explain metabolic perturbations observed following ClpP activation, including upregulation in the serine biosynthetic pathway and loss of cellular aspartate pools.

ALAS1, the rate limiting step of heme biosynthesis, has previously been identified as a ClpP substrate (Kubota et al., 2016; Whitman et al., 2018; Wong and Houry, 2019) and loss of heme biosynthesis has been implicated as a metabolic vulnerability in acute myeloid leukemia (Lin et al., 2020). Our data clearly showed significant and rapid downregulation of ALAS1 in response to ClpP activators. Because LonP has also been reported to degrade ALAS1 (Tian et al., 2011; Kubota et al., 2016), we additionally utilized bortezomib co-treatment and LonP siRNA to determine the role of LonP in ALAS1 stability following treatment with ONC201 or TR-57 (Figure 6). We found ALAS1 was stabilized in ClpP-KO SUM159 cells, but not in either bortezomib-treated or LonP knockdown SUM159 cells, suggesting that loss of ALAS1 is due to ClpP activation in these experiments. We attempted to investigate whether loss of the heme biosynthetic pathway was essential for ClpP activation-mediated cell growth inhibition. However, supplementation with hemin, a cell permeable form of heme, did not restore cell growth in treated WT SUM159 cells, suggesting loss of the heme biosynthetic pathway may be one of many consequences of ClpP activation that leads to reduced cell growth. Inability to sensitize or desensitize cells to ONC201 or TR-57 treatment could be due to ClpP activation broadly affecting a number of metabolic pathways in cancer cells. Therefore, interrupting or restoring single metabolic pathways may not be sufficient to increase or decrease sensitivity to ClpP activators.

In summary, this study has identified significant proteomic, transcriptomic, and metabolomic perturbations occurring following ClpP activation by two chemically distinct ClpP agonists. We have shown that the vast majority of these changes are ClpP-dependent, including induction of ATF4-dependent genes and protein-level loss of the heme biosynthetic pathway. Applying multi-omics analysis identified 42 significantly perturbed pathways (Supplementary Data S7) following treatment with ONC201 or TR-57, including amino acid biosynthesis, TCA cycle, and the citrulline pathway. The identification of the broad effects of ClpP activation on cancer cell metabolism provides critical insight into the mechanism by which pharmacological activation of ClpP inhibits cancer cell growth and suggest a response that is both multi-nodal and dependent on the dysregulation of multiple pathways.

## Materials and methods

### Cell culture

The human triple-negative breast cancer (TNBC) cell line SUM159 was cultured in Dulbecco’s modified Eagle’s medium: Nutrient Mixture F-12 (DMEM/F-12, Gibco, 11,330–032) supplemented with 5% fetal bovine serum (VWR-Seradigm, 97,068–085), 1% antibiotic/antimycotic (ThermoFisher Scientific, 15,240,062), 5 μg/ml insulin (Gibco, 12,585,014), and 1 μg/ml hydrocortisone. CRISPRi was used to generate CLPP-knockout SUM159 cells as previously described (Fennell et al., 2022). WT MDA-MB-231 cells were cultured in RPMI 1640 media (Gibco, 11,875–093) supplemented with 10% FBS and 1% antibiotic/antimycotic. Cells were incubated at 5% CO_2_ and 37 °C and periodically tested for *mycoplasma*.

### Cell viability

#### Total cell counting

WT and ClpP-KO SUM159 cells were seeded at 1,000 cells/well in a 96-well plate (Perkin Elmer, 6,005,050) and allowed to adhere overnight. Media was replaced by 100 μL of media supplemented with drugs at concentrations indicated in figures and figure legends. 0.1% DMSO treatment was used a negative control. Following treatment, media was aspirated and replaced with 100 μl Dulbecco’s phosphate buffered saline (DBPS, Gibco, 14,190–144) with 1 μg/mL Hoechst stain (ThermoFischer Scientific, H3570) and allowed to incubate at 37 °C for 30 min. Cell number was quantified using Celigo Imaging Cytometer (Nexcelom).

#### Crystal violet assays

Crystal violet assays were performed by plating WT or ClpP-KO SUM159 or WT MDA-MB-231 cells (1,000 cells/well) in a 6-well plate (Corning) and allowing them to adhere overnight. Media was aspirated and replaced by 2 ml of media containing desired compound treatments. Media was replaced with growth media containing the same drug treatments every 48 h until one well was 100% confluent. Once confluent, cells were stained (0.5% crystal violet, 20% methanol) for 10 min at RT, rinsed 3 times and allowed to dry overnight.

### RNAi

All siRNA stocks were purchased through Horizon Discovery at 2 nmol/siRNA and resuspended in 100 ul 1X siRNA reconstitution buffer (Horizon Discovery) to create a 20 μM stock. Dharmafect I was diluted 1:266 in OptiMEM media and separated into the appropriate number of 250 μl aliquots. Individual siRNAs were added to OptiMEM:Dharmafect aliquots for a final concentration of 125 nM, incubated at RT for 30 min, and then added to a 6-well plate. SUM159 cells were washed with DPBS and trypsinized and 37 °C for 5 min. Cells were resuspended to a final concentration of 1 × 10^5^ cells/ml in 1:1 DMEM:F-12 supplemented with 5% FBS, 5 μg/mL insulin, and 1 μg/ml hydrocortisone. Cells were added to each well (1.0 ml/well) containing siRNA and incubated for 24 h. Cells were then treated with 10 μM ONC201 or 150 nM TR-57 for the timepoints indicated in the figure legends and harvested as described above.

### Proteomics

#### Sample preparation

For whole cell and phosphoproteomics, SUM159 (WT and ClpP-KO) cells were plated in a 10 cm^2^ dish (Corning) and allowed to adhere overnight. Cells were then treated with 0.1% DMSO, 10 μM ONC201, or 150 nM TR-57 for 24 h. Cells were washed with 3 × 5 mL ice-cold Dulbecco’s Phosphate Buffered Saline (DPBS, Gibco, 14,190–144) and lysed in 8M urea buffer [8M urea, 50 mM Tris (pH 7.4), 2.5 mM Na_3_VO4, 1 mM NaF, 1X protease inhibitor cocktail, 1X phosphatase inhibitor cocktail 2, 1X phosphatase inhibitor cocktail 3]. Lysates were clarified by centrifugation and protein concentration quantified by Bradford Assay (BioRad). Protein lysates (400 μg) were reduced with 5 mM DTT at 56°C for 30 min, then alkylated with 15 mM iodoacetamide at RT in the dark for 45 min. Protein was precipitated using 4-times the volume of cold acetone and stored overnight at −20°C. The next day, samples were centrifuged at 15,000 x g for 15 min at 4°C, then the protein pellets were reconstituted in 1M urea. Samples were digested with LysC (Wako) for 2 h and trypsin (Promega) overnight at 37°C at a 1:50 enzyme:protein ratio. The resulting peptide samples were acidified, desalted using Thermo desalting spin columns, then the eluates were dried *via* vacuum centrifugation. Peptide concentration was determined using Pierce Quantitative Fluorometric Peptide Assay.

Samples were split into two TMTpro 16plex sets based on drug treatment (ONC201 or TR-57). In TMT set 1, the samples included: SUM159 WT cell line with 3 DMSO controls (n = 3) and ONC201 treated samples (n = 4), and SUM159 ClpP KO cell line with 3 DMSO controls (n = 3) and ONC201 treated samples (n = 4), along with two pooled samples to assess technical variability. TMT set 2 included the same experimental design except with TR57 treatment instead of ONC201. A total of sixteen samples per set, which were labeled with TMTpro 16plex (Thermo Fisher); a sample key can be found in the PRIDE submission (PXD038990). 125 μg of each sample was reconstituted with 50 mM HEPES pH 8.5, then individually labeled with 250 µg of TMTpro reagent for 1 h at room temperature. Prior to quenching, the labeling efficiency was evaluated by LC-MS/MS analysis of a pooled sample consisting of 1ul of each sample. After confirming >98% efficiency, samples were quenched with 50% hydroxylamine to a final concentration of 0.4%. Labeled peptide samples for each set were combined 1:1, desalted using Thermo desalting spin column, and dried *via* vacuum centrifugation. The dried TMT-labeled samples (two TMT sets total) were fractionated using high pH reversed phase HPLC (Mertins et al., 2018). Briefly, the samples were offline fractionated over a 90 min run, into 96 fractions by high pH reverse-phase HPLC (Agilent 1,260) using an Agilent Zorbax 300 Extend-C18 column (3.5-µm, 4.6 × 250 mm) with mobile phase A containing 4.5 mM ammonium formate (pH 10) in 2% (vol/vol) LC-MS grade acetonitrile, and mobile phase B containing 4.5 mM ammonium formate (pH 10) in 90% (vol/vol) LC-MS grade acetonitrile. The 96 resulting fractions were then concatenated in a non-continuous manner into 24 fractions and 5% of each were aliquoted, dried down *via* vacuum centrifugation and stored at −80°C until further analysis.

The remaining 95% of each fraction was further concatenated into 3 fractions and dried down *via* vacuum centrifugation. For each fraction, phosphopeptides were enriched with a two-step method, where High Select Fe-NTA kit (Thermo) was first used, and from that enrichment step the flow-through and eluates were collected. Then, with the flow-through of each fraction, the High Select TiO_2_ kit (Thermo) was used to further enrich for phosphopeptides. Manufacturer protocols were followed for both enrichments. Fe-NTA and TiO_2_ eluates were dried down *via* vacuum centrifugation and stored at −80°C until further analysis.

#### Data acquisition and processing

Two sets of 24 fractions for the proteome analysis and two sets of six fractions (FeNTA and TiO_2_ eluates) for the phosphoproteome analysis were analyzed by LC/MS/MS using an Easy nLC 1,200 coupled to an Orbitrap Fusion Lumos Tribrid mass spectrometer (Thermo Scientific). Samples were injected onto an Easy Spray PepMap C18 column (75 μm id × 25 cm, 2 μm particle size) (Thermo Scientific) and separated over either a 120-min method for the proteome fractions or a 150-min method for the phosphoproteome fractions. For the proteome fractions, the gradient for separation consisted of 5%–40% mobile phase B at a 250 nl/min flow rate, where mobile phase A was 0.1% formic acid in water and mobile phase B consisted of 0.1% formic acid in 80% ACN.

For the proteome fractions, the Lumos was operated in SPS-MS3 mode (McAlister et al., 2014) with a 3s cycle time. Resolution for the precursor scan (m/z 400–1,500) was set to 120,000 with a AGC target set to standard and a maximum injection time of 50 m. MS2 scans consisted of CID normalized collision energy (NCE) 32; AGC target set to standard; maximum injection time of 50 m; isolation window of 0.7 Da. Following MS2 acquisition, MS3 spectra were collected in SPS mode (10 scans per outcome); HCD set to 55; resolution set to 50,000; scan range set to 100–500; AGC target set to 200% with a 100 m maximum inject time. Dynamic exclusion was set to 30 s.

For the phosphoproteome fractions, the Lumos was operated in MS2 mode (Hogrebe et al., 2018; Klann et al., 2020) with a 3s cycle time. Resolution for the precursor scan (m/z 400–1,500) was set to 60,000 with a AGC target set to standard and a maximum injection time of 50 m. For MS2 scans, HCD was set to 35; AGC target set to 200%; maximum injection time of 120 m; isolation window of 0.7 Da; resolution set to 50,000. Dynamic exclusion was set to 30 s.

##### Identification and annotation

For proteome and phosphoproteome data, all raw files were processed using Proteome Discoverer version 2.5. “TMTpro 16plex” was used as the quantitation method, and the two TMTpro 16plex datasets (Set 1: ONC201; Set 2: TR-57) were analyzed separately. Peak lists were searched against a reviewed Uniprot human database (downloaded February 2020 containing 20,350 sequences), appended with a common contaminants database (from MaxQuant, containing 245 sequences), using Sequest HT within Proteome Discoverer. Data were searched with up to two missed trypsin cleavage sites and fixed modifications were set to TMTpro peptide N-terminus and Lys, and carbamidomethyl Cys. Dynamic modifications were set to N-terminal protein acetyl and oxidation Met. Percolator node was used to calculate peptide false discovery rates (FDR).

For phosphoproteome data, additional dynamic modification was set to phosphorylation Ser, Thr, Tyr. TMT quantitation was set to MS2, precursor mass tolerance was set to 10 ppm and fragment mass tolerance was set to 0.02 Da. Peptide FDR was set to 1%. The ptmRS node was used to localize phosphorylation sites within peptides. Reporter abundance based on intensity and co-isolation threshold was set to 50. Normalization was enabled (‘use all peptides’).

For proteome data, quantitation was set to MS3, precursor mass tolerance was set to 20 ppm and fragment mass tolerance was set to 0.5 Da. Peptide FDR was set to 1%. Reporter abundance based on intensity, SPS mass matches threshold set to 50, and razor and unique peptides were used for quantitation. Normalization was enabled (‘use all peptides’).

### Transcriptomics

#### Sample preparation

SUM159 (WT and ClpP-KO) cells were plated and treated as described for Proteomics. Cells were washed 3 × 5 ml ice-cold DPBS and RNA was extracted using a Qiagen RNeasy Kit (74,104) following manufacturer’s directions. RNA samples were sent to Novogene Co. for RNAseq analysis.

#### Identification and annotation

Raw reads for each sample were obtained as FASTQ files from Novogene Co. And was indexed and quantified in alignment-based mode through Salmon using reference transcriptome [Transcript Sequences v. 27] from GENCODE. Resulting quantification files were imported to R and DESeq2 was used to normalize samples and analyze differential expression. Significance cutoffs including a base mean exceeding 50 and *p*-value equal to or exceeding 0.5 were applied to the dataset.

### Metabolomics

#### Sample preparation

SUM159 (WT and ClpP-KO) cells were plated as described for proteomics analysis. Cells were washed 3 × 5 ml ice-cold DPBS and metabolites were extracted as previously described (Stewart et al., 2016; Winnike et al., 2018). Briefly, 1 ml −20 °C acetonitrile and 750 μl ice cold H_2_O were added to washed plates and cells were mechanically scraped and transferred to 15 ml conical tubes and stored at −80 °C until extraction was performed. To extract metabolites, ∼five 2 mm zirconia beads were added to each 15 ml conical followed by 500 μl chloroform (−20 °C) and samples were vortexed 3 × 30 s. Samples were then centrifuged in a 4 °C swing-bucket centrifuge (3,700*g*, 60 min). The aqueous layer was then transferred to a 2 mL Lo-Bind tube and the organic layer to a glass vial. Remaining samples were transferred to a 1.5 mL Lo-Bind tube and 15 ml conicals were washed with 300 μl 2:1 chloroform:methanol solution (−20 °C) and transferred to corresponding 1.5 ml Lo-Bind tube and centrifuged at 4 °C (15,000*g*, 20 min). Aqueous and organic layers were transferred to corresponding 2 ml Lo-Bind tube and glass vial for each sample and frozen at −80 °C.

Aqueous fractions of cell extracts were dried by speed vac and reconstituted using 200 μl of reconstitution solution (95:5 water:methanol), and 150 μl of the reconstituted extract was transferred to new tubes. An aliquot of 20 μl was taken from each extract and combined to make a total study pool (SP). All samples and the SP were centrifuged at 4 °C and 16,000 x g for 10 min, and the supernatants were transferred to LC-MS vials. An injection volume of 5 μl was used for LC-MS analysis.

#### Data acquisition and processing

Metabolomics data were acquired on a Vanquish UHPLC system coupled to a Q Exactive™ HF-X Hybrid Quadrupole-Orbitrap Mass Spectrometer (Thermo Fisher Scientific, San Jose, CA). Metabolites were separated *via* an HSS T3 C18 column (2.1 × 100 mm, 1.7 µm, Waters Corporation) at 50 °C with binary mobile phase of water (A) and methanol (B), each containing 0.1% formic acid (v/v). The UHPLC linear gradient started from 2% B, and increased to 100% B in 16 min, then held for 4 min, with the flow rate at 400 μl/min. The untargeted data was acquired from 70 to 1,050 m/z using the data-dependent acquisition mode. Method blanks and SP injections were placed after every six samples (n = 3 each). Progenesis QI (version 2.1, Waters Corporation) was used for peak picking, alignment, and normalization. Background signals were filtered out by removing peaks with a fold change less than 3 in the total SP vs. the blank injections. Samples were then normalized in Progenesis QI using the “normalize to all” feature (Välikangas et al., 2018). Coefficient of variation (CV) values were calculated across the total SP replicates for each peak and those with CV >30% were removed. Filtered, normalized data was exported and multivariate analysis was performed using SIMCA 16.

#### Identification and annotation

Metabolites were identified in Progenesis QI using an in-house physical standards library of >2,400 reference standards. All reference standards were analyzed under the same instrument conditions used to analyze the study samples. Peaks were matched to metabolites in the in-house library by exact mass (MS), fragmentation pattern (MS/MS), and retention time (rt). An ontology system was provided for each peak match to indicate the evidence basis for each identification. A peak was considered to have a match by rt if the peak eluted within 0.5 min compared to the reference standard, an MS match was defined as < 5 ppm error compared to the theoretical mass based on the metabolite chemical formula, and an MS/MS match was defined as a similarity score >30% to a reference standard (calculated using MS/MS match algorithms in Progenesis QI). Peaks were also annotated to additional metabolites by matching signals to public mass spectral databases (NIST, METLIN, HMDB). OL1 refers to an in-house metabolite match by rt, MS, and MS/MS; OL2a refers to an in-house match by rt and MS only; OL2b refers to an in-house match by MS and MS/MS only; PDa refers to a public database match by MS and experimental MS/MS, PDb refers to a public database match by MS and theoretical MS/MS, PDc refers to a public database match by MS and isotope similarity (>90%); PDd refers to a public database match by MS only.

### Immunoblotting

SUM159 cells (WT and ClpP-KO) were plated (1 × 10^5^ cells/well) on a 6-well plate (Corning, 3,516) and incubated with compounds at concentrations as indicated in figure legends. Following treatment, cells were rinsed 3 × with 2 ml of ice-cold DPBS and lysed using RIPA buffer (20 mM Tris [pH 7.4], 137 mM NaCl, 10% glycerol, 1% Nonidet P-40, 0.5% deoxycholate, 2 mM EDTA) supplemented with 10 mM NaF, 2 mM Na_3_VO_4_, 0.0125 μM calyculin A, and complete protease inhibitor cocktail (Roche Diagnostics, 11,873,580,001). Lysates were clarified and immunoblotted as previously described. Membranes were incubated with primary antibodies [ATF4 (Cell Signaling Technologies, 11,815), ASNS (Cell Signaling Technologies, 20,843), ALAS1 (Abcam, ab154860), LonP (Cell Signaling Technologies, 28,020), ClpP (Cell Signaling Technologies, 14,181), Vinculin (Santa Cruz Biotechnology sc-73614)] diluted 1:1,000 in 1% fish gelatin (Sigma Aldrich, G7041)/TBST overnight at 4 °C, then washed 3 × 5 min in TBST and incubated at room temperature in the appropriate secondary antibody (1:10,000 in 5% milk/TBST) for 1 h. Membranes were then washed 3 × 5 min in TBST and incubated in ECL reagent (BioRad, 170–5,061) and imaged using Chemidoc MP (BioRad). Acquired images were processed using ImageLab software (BioRad).

### Statistics and software

ImageLab (BioRad) was used to process immunoblot images. Prism 9 (GraphPad) was used to generate bar charts. BioVenn (Hulsen et al., 2008) was used to generate Venn diagrams and VolcanoseR (Goedhart and Luijsterburg, 2020) was used to generate volcano plots. Proteome Discoverer was used to perform statistical analysis on proteomics and phosphoproteomics data. Protein and phosphopeptide *p*-values for each pairwise comparison was calculated using Student’s t-test, and *p*-value <0.05 was considered significant. Log_2_(Fold Change) was calculated for each pairwise comparison using the normalized TMT intensities averaged across 3 replicates. Salmon and DESeq were used to process transcriptomics data. *p*-values for each pairwise comparison was calculated using Student’s t-test, and *p*-value <0.05 was considered significant. *p*-values were not adjusted for multiple comparisons for use in volcano plots due to the exploratory, rather than confirmatory, nature of this study (Bender and Lange, 2001). Adjusted *p*-values are shown for proteomic and transcriptomic data in Figure 5, Supplementary Figure 6S2. Log_2_(Fold Change) was calculated for each pairwise comparison using normalized counts averaged across 3 replicates ProgenesisQI was used to process UPLC-MS metabolomics data and SIMCA (Sartorious) was used to calculate variable importance to projection (VIP) scores. Log_2_(Fold Changes) of means (proteins, transcripts) or medians (metabolomics) and their respective -log_10_ (*p*-value) were determined for significant proteins, transcripts, and metabolites using Excel (Microsoft). Proteomic and transcriptomic observations meeting the fold change and significance threshold (Log_2_(Fold Change)≥|0.5| and -log_10_ (*p*-value) ≤-1.3) and metabolomic observations meeting the fold change threshold, significance threshold, or SIMCA VIP score ≥0.95 were used for multi-omics analysis. These thresholds were chosen to due frequent use in -omics studies. MetaCore pathway analysis software (Clarivate Analytics) was used for multi-omics data analysis. DAVID (Huang et al., 2009) (v. 2021) was used for gene ontology analysis. Specifically, data was analyzed using GO terms for biological processes (GOTERM_BP_DIRECT), cellular components (GOTERM_CC_DIRECT), molecular functions (GOTERM_MF_DIRECT), and KEGG pathways. Data collected from DAVID is available in Supplementary Material. BioRender was utilized to generate all schematics and pathways.

## Data Availability

The original contributions presented in the study are publicly available. Proteomics data is available on the Proteomics Identification Database (PRIDE, https://www.ebi.ac.uk/pride/; Project ID: PXD038990). Transcriptomics data is available at the NCBI’s Gene Expression Omnibus (GEO, https://www.ncbi.nlm.nih.gov/geo/; Series accession number: GSE221327). Metabolomics data is available at the NIH Common Fund’s National Metabolomics Data Repository (NMDR) website (Metabolomics Workbench, https://www.metabolomicsworkbench.org; Project ID: PR001550; DOI: http://dx.doi.org/10.21228/M8BM6G). All other data is available upon request to corresponding author.

## Abbreviations

ADEP, acyldepsipeptide; ATF4, activating transcription factor 4; ClpP, mitochondrial matrix caseinolytic protease, proteolytic subunit; ClpX; mitochondrial matrix caseinolytic peptidase, chaperone subunit X; GO, gene ontology; ISR, integrated stress response; LC-MS, liquid chromatography-mass spectrometry; mtDNA, mitochondrial DNA; OXPHOS, oxidative phosphorylation; TCA, tricarboxylic acid; TNBC, triple-negative breast cancer; TRAIL, TNF-related apoptosis-inducing ligand; UPR^mt^, mitochondrial unfolded protein response.
