# Supplementary figures and images for: Multi-omics analyses reveal ClpP activators disrupt essential mitochondrial pathways in triple-negative breast cancer
Source: Front Pharmacol. 2023 Mar 31;14:1136317. doi: 10.3389/fphar.2023.1136317 (PMC10103842; doi:10.3389/fphar.2023.1136317)

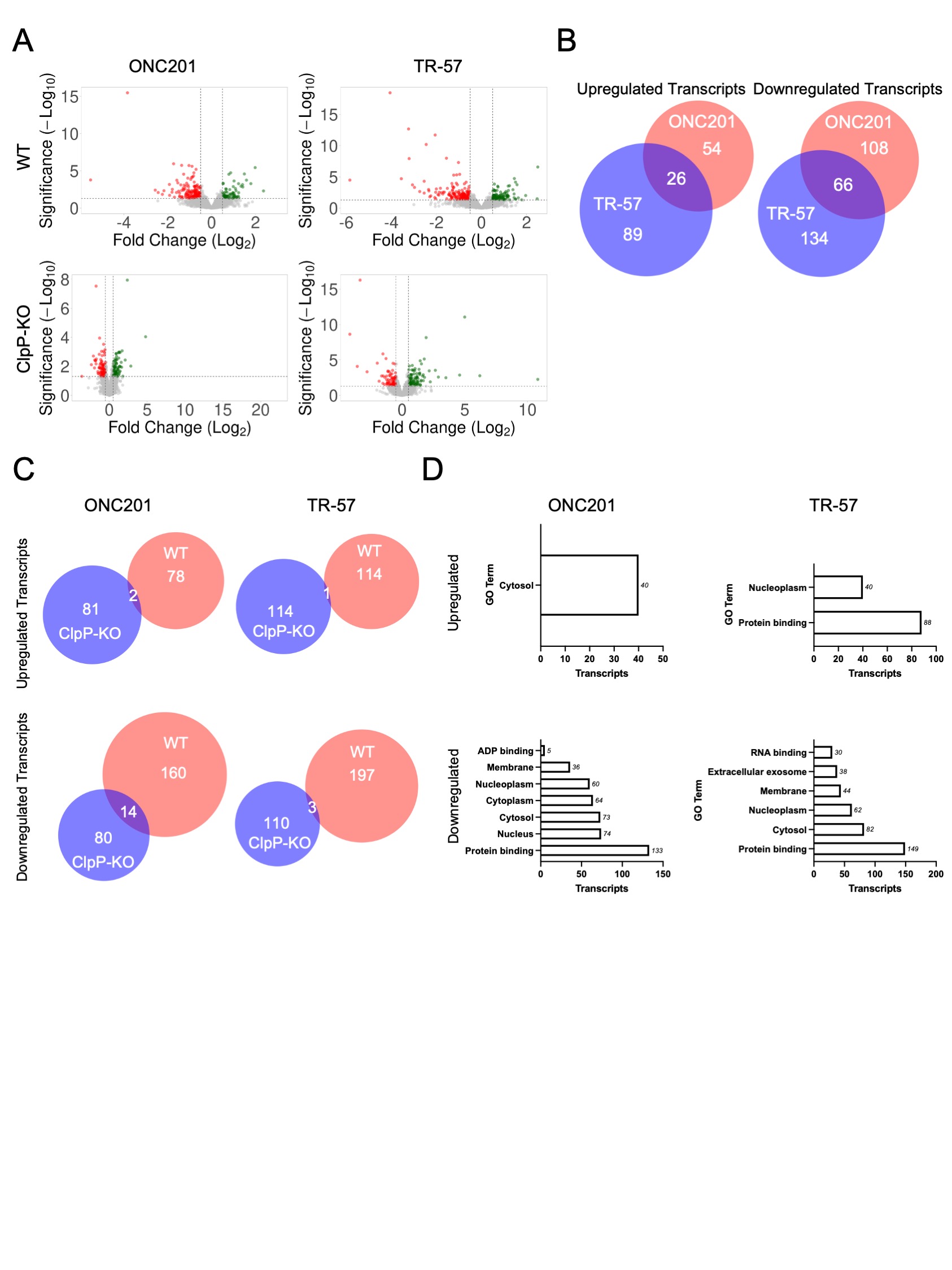

Supplement: Supplementary file 1 [file Image3.jpg]

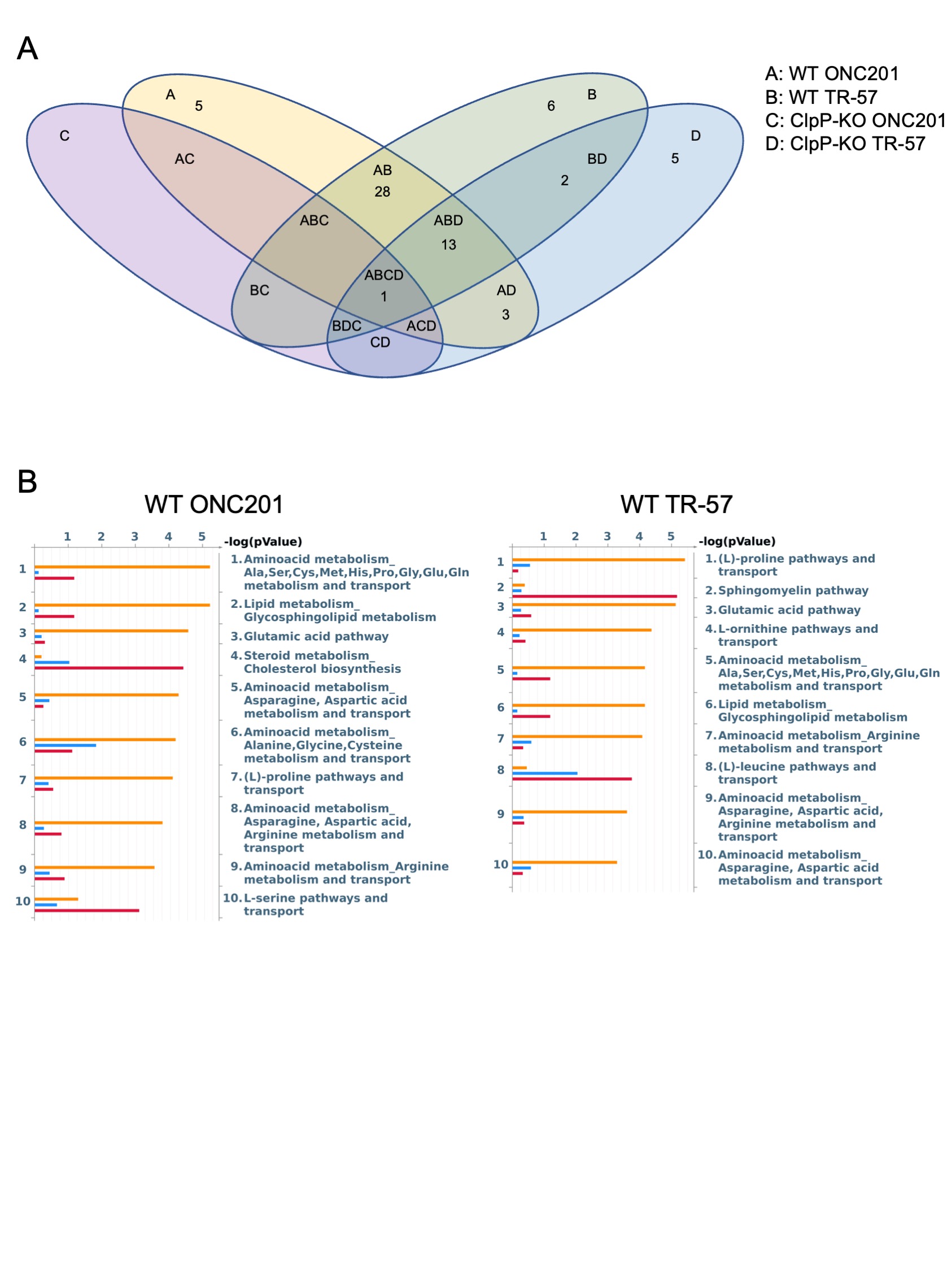

Supplement: Supplementary file 5 [file Image4.JPEG]

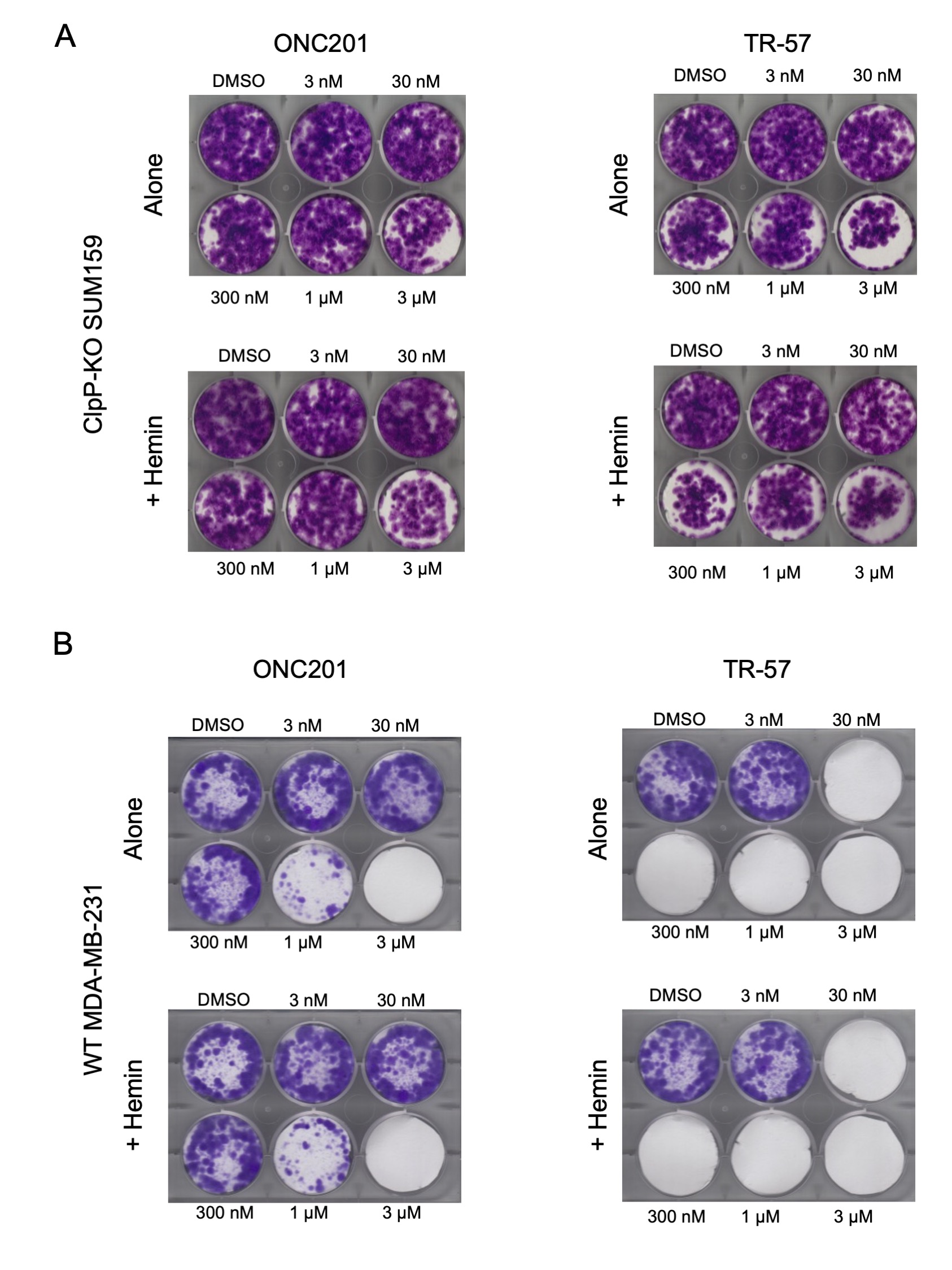

Supplement: Supplementary file 6 [file Image7.JPEG]

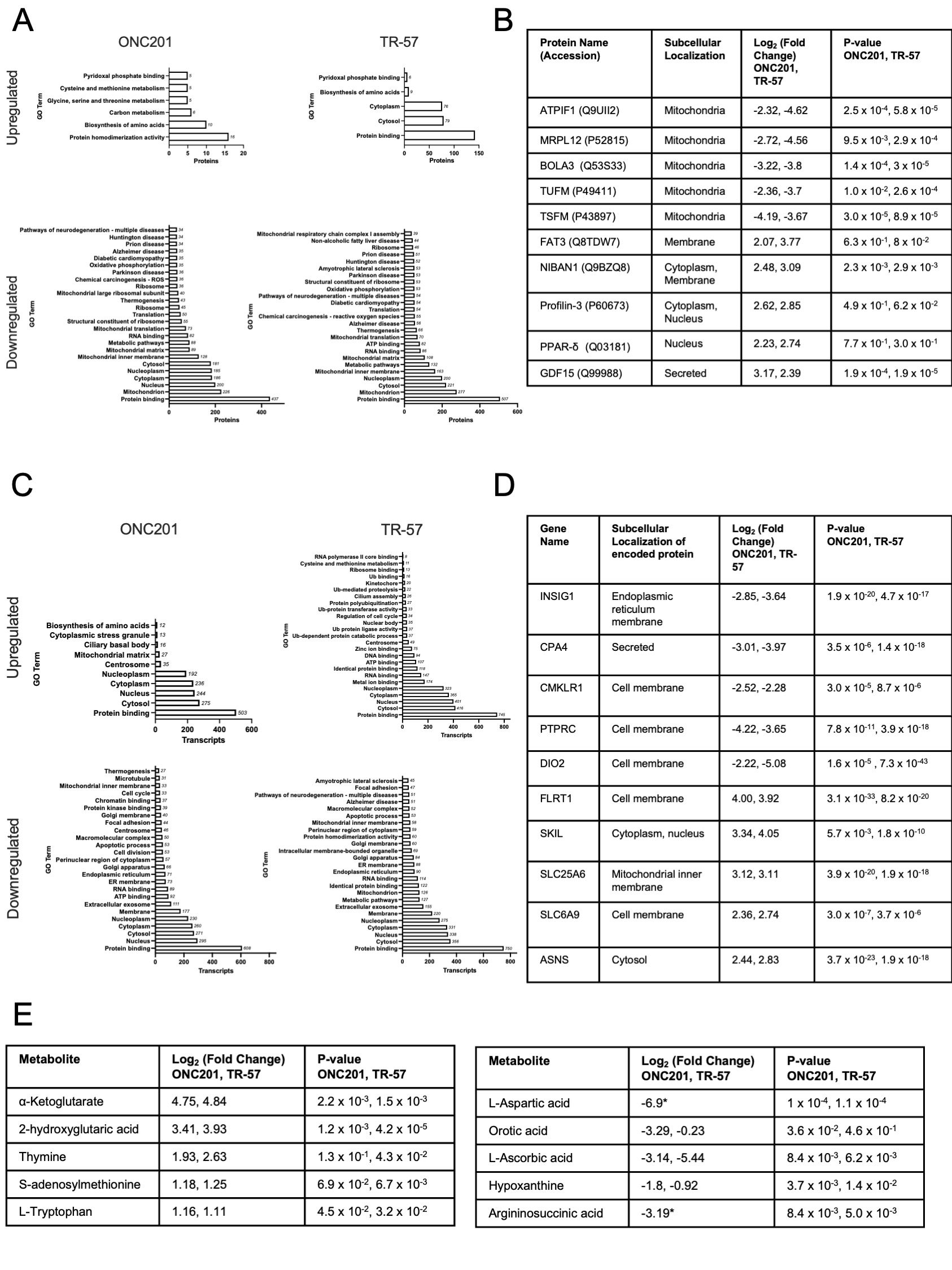

Supplement: Supplementary file 7 [file Image2.JPEG]

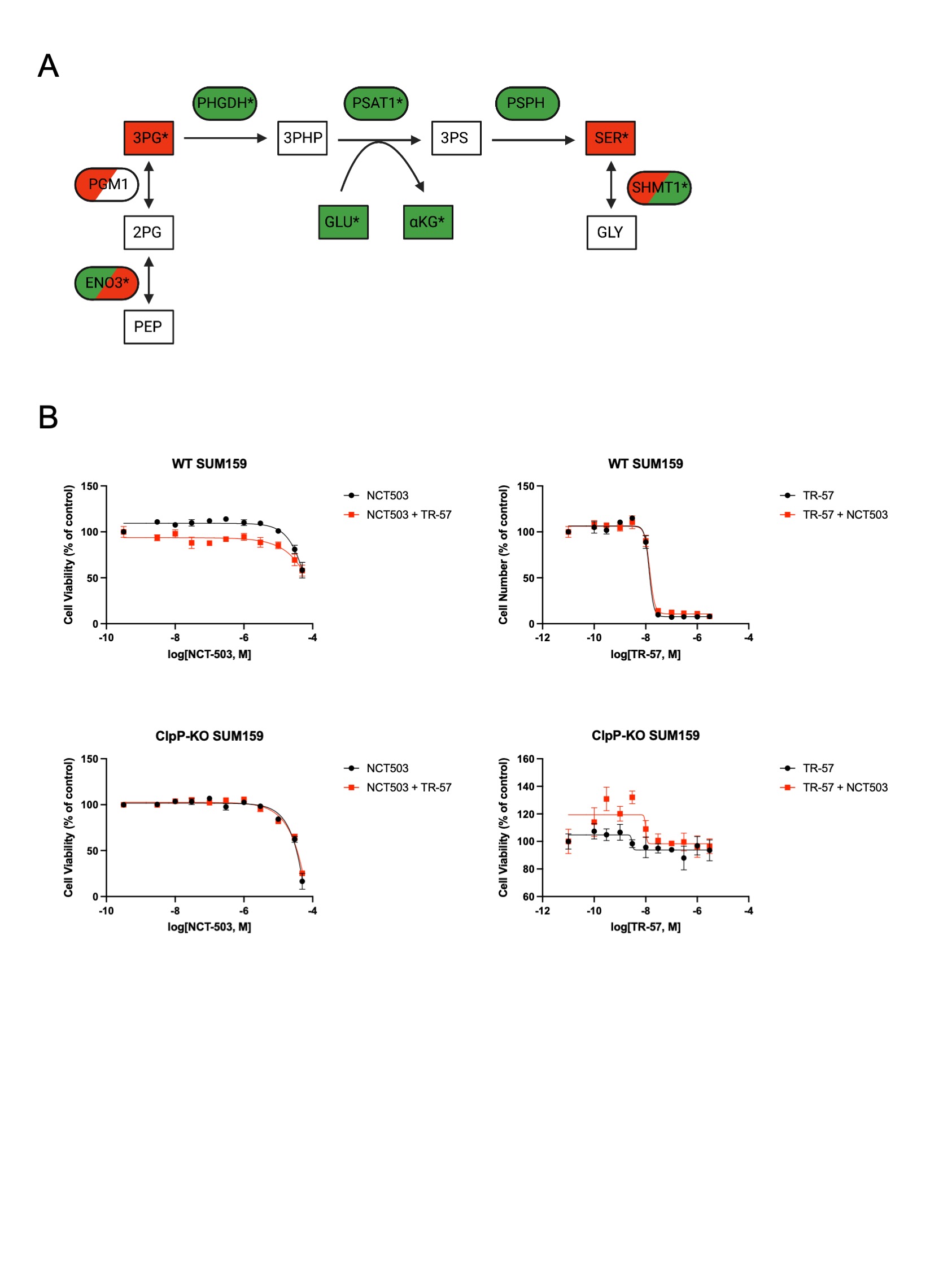

Supplement: Supplementary file 8 [file Image5.JPEG]

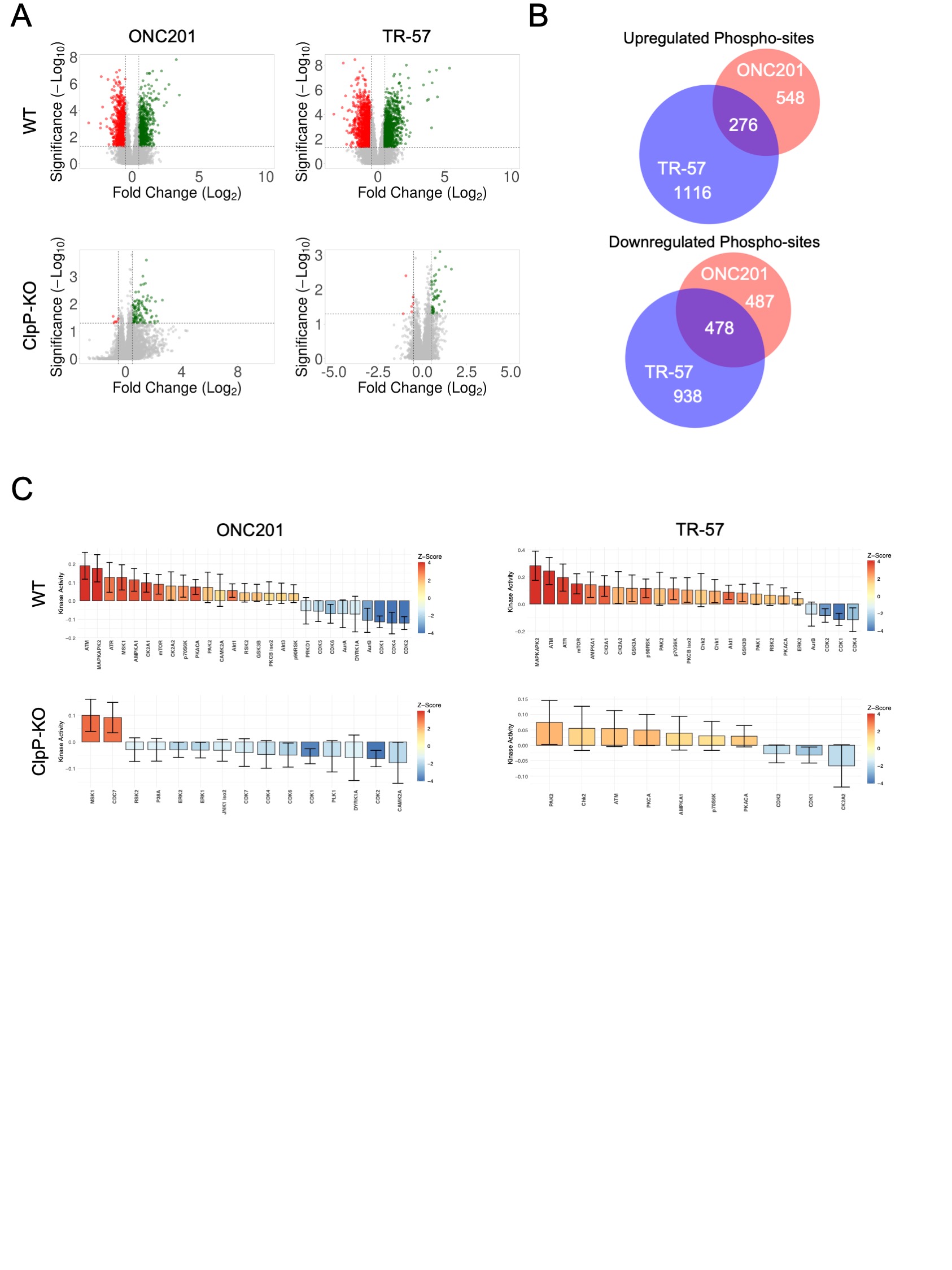

Supplement: Supplementary file 11 [file Image1.jpg]

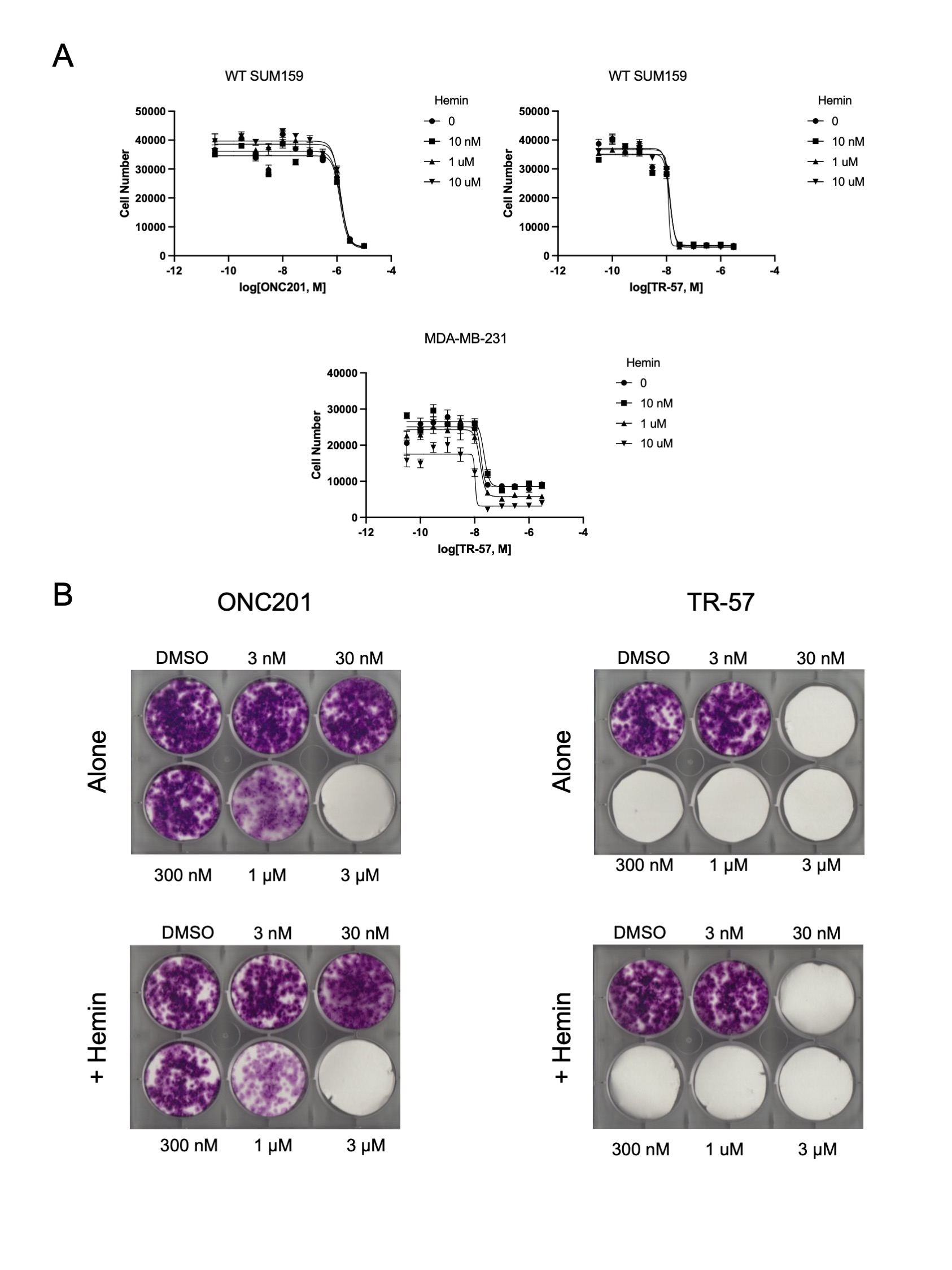

Supplement: Supplementary file 12 [file Image6.JPEG]
